# SKP1-like protein, CrSKP1-e, interacts with pollen-specific F-box proteins and assembles into SCF-type E3 complex in ‘Wuzishatangju’ (*Citrus reticulata* Blanco) pollen

**DOI:** 10.7717/peerj.10578

**Published:** 2020-12-22

**Authors:** Yi Ren, Qingzhu Hua, Jiayan Pan, Zhike Zhang, Jietang Zhao, Xinhua He, Yonghua Qin, Guibing Hu

**Affiliations:** 1 State Key Laboratory for Conservation and Utilization of Subtropical Agro-bioresources/ Guangdong Provincial Key Laboratory of Postharvest Science of Fruits and Vegetables/Key Laboratory of South China Horticultural Crop Biology and Germplasm Enhancement, College of Horticulture, South China Agricultural University, Guangzhou, China; 2 State Key Laboratory for Conservation and Utilization of Subtropical Agro-bioresources, College of Agriculture, Guangxi University, Nanning, China

**Keywords:** *Citrus retuculata* Blanco, Self-incompatibility, SCF complex, F-box, SKP1-like, Cullin1

## Abstract

S-ribonuclease (S-RNase)-based self-incompatibility (SI) mechanisms have been extensively studied in Solanaceae, Rosaceae and Plantaginaceae. S-RNase-based SI is controlled by two closely related genes, *S-RNase* and *S-locus F-box* (*SLF*), located at a polymorphic S-locus. In the SI system, the SCF-type (SKP1-CUL1-F-box-RBX1) complex functions as an E3 ubiquitin ligase complex for ubiquitination of non-self S-RNase. Pummelo (*Citrus grandis*) and several mandarin cultivars are suggested to utilize an S-RNase-based SI system. However, the molecular mechanism of the non-S-factors involved in the SI reaction is not straightforward in *Citrus*. To investigate the SCF-type E3 complex responsible for the SI reaction in mandarin, SLF, SKP1-like and CUL1 candidates potentially involved in the SI reaction of ‘Wuzishatangju’ (*Citrus reticulata* Blanco) were identified based on the genome-wide identification and expression analyses. Sixteen pollen-specific *F-box* genes (*CrFBX1-CrFBX16*), one pollen-specific *SKP1-like* gene (*CrSKP1-e*) and two *CUL1* genes (*CrCUL1A* and *CrCUL1B*) were identified and cloned from ‘Wuzishatangju’. Yeast two-hybrid (Y2H) and in vitro binding assays showed that five CrFBX proteins could bind to CrSKP1-e, which is an ortholog of SSK1 (SLF-interacting-SKP1-like), a non-S-factor responsible for the SI reaction. Luciferase complementation imaging (LCI) and in vitro binding assays also showed that CrSKP1-e interacts with the N-terminal region of both CrCUL1A and CrCUL1B. These results indicate that CrSKP1-e may serve as a functional member of the SCF-type E3 ubiquitin ligase complex in ‘Wuzishatangju’.

## Introduction

Self-incompatibility (SI) promotes genetic diversity and inhibits inbreeding depression by recognizing self (or genetically related) pollen (Franklin-Tong, 2008). In S-RNase-based SI system, the recognition specificity is regulated by a highly polymorphic locus, named *S-RNase* and multiple *SLFs*. The S-RNase is abundant in style cells, and then secretes into the stylar canal to infiltrate into the pollen tube, where it produces cytotoxin in an S-haplotype independent manner (Luu et al., 2000; McClure et al., 1990). *SLFs* are specifically expressed in pollen and serve as pollen determinant in SI system (Kubo et al., 2010). The mechanisms of S-RNase-based SI reaction have been extensively investigated in Rosaceae, Solanaceae and Plantaginaceae, and some species of Rutaceae and Rubiaceae (Nowak et al., 2011; Zhang et al., 2018; Liang et al., 2020).

‘Self-recognition’ and ‘non-self-recognition’ systems can be used to understand SI mechanism (Fujii, Kubo & Takayama, 2016). Pollen-specific *SFB* (S-haplotype specific F-box) is a single S pollen gene which specifically links with *S-RNase* (Ushijima et al., 2003). In the system, non-self S-RNase is inactivated by a ‘general inhibitor’, an F-box protein encoded by *SLFL* (S-locus F-box like) which is also linked to *S-RNase*. The activity of self S-RNase is specifically protected by SFB, leading to RNA degradation of self-pollen tube and growth inhibition (Ushijima et al., 2004). In a ‘non-self-recognition’ system, the pollen determinants are regulated by multiple S-locus *F-box* genes, termed as SLF in Solanaceae and Plantaginaceae and SFBB (S-haplotype specific F-box brothers) in Maloideae. In general, multiple types of *SLF/SFBB* with intrahaplotypic diversity are linked with *S-RNase* within a haplotype. Each type of SLF/SFBB can recognize and degrade a subset of non-self S-RNase proteins via the ubiquitination pathway (Kakui et al., 2011; Kubo et al., 2010).

In S-RNase-based reaction, the F-box protein acts as an acceptor of the canonical SCF complex (E3 ubiquitin ligase), and coordinates ubiquitination of non-self S-RNase with degradation by 26S proteasome (Hua & Kao, 2006; Entani et al., 2014). The role of S-locus *F-box* gene in SI has been confirmed by genotype-dependent transformation in vivo, where the F-box targets non-self S-RNase and degrades it (Sijacic et al., 2004). In Rosaceae, pollen-part mutant of S-locus which confers the self-compatibility reaction by encoding a non-functional F-box protein can be used to identify pollen factor (Hauck et al., 2006; Ushijima et al., 2004). However, no typical amino acid patterns for S-locus F-box proteins have been reported. In addition to F-box domain, S-locus F-box proteins also contain a FBA domain at the C-terminal region. In *Arabidopsis*, 92 non-S-locus F-box containing a FBA domain called SLF-like (SFL) was identified (Wang et al., 2004). In apple, all SFBBs contain FBA domain in the C-terminus (Minamikawa et al., 2010). Therefore, FBA domain can be used as a trait to characterize S-locus F-box candidates. Additionally, S-locus *F-box* genes show pollen-specific expression (Chen et al., 2018a; Zhao et al., 2002). *SFBBs* commonly cluster with the *S-RNase* in apple genome by S-locus deciphering (Minamikawa et al., 2010). Intra- (Inter-) haplotype sequence divergences of S-locus F-box proteins are considered to be an important characteristic to recognize various allelic polymorphic S-RNase proteins (Fujii, Kubo & Takayama, 2016; Kakui et al., 2011). So far, the S-locus *F-box* candidate genes have not been elucidated in ‘Wuzishatangju’.

Ubiquitin-dependent proteolysis contributes to detoxification caused by SCF-type E3 ligase in S-RNase-based SI reaction. Ubiquitin targeting is achieved by an ATP-dependent consecutive reaction of a ubiquitin activating enzyme (E1), ubiquitin conjugating enzymes (E2) and ubiquitin ligase (E3). At the end of the cascade, the E3 ligase confers specificity for substrate recognition and brings the target to E2 for ubiquitin conjunction (Schwechheimer, 2018). For the canonical SCF-type E3 ligase complex, the CUL1 protein acts as a scaffold that adopts a stalk-like structure to interact with an F-box protein through the SKP1 protein adaptor in N-terminal region and binds RBX1 on the other end. F-box subunit recognizes the protein substrate via the C-terminal region which exhibits high diversity (Ning et al., 2016; Zimmerman, Schulman & Zheng, 2010). The *SKP1-like* gene involved in S-RNase-based SI reaction has been identified. A pollen specific *AhSSK1* encoding an SKP1-like protein was first identified in *Antirrhinum hispanicum*, and its interaction with S-locus F-box protein was confirmed by Y2H and GST-pull down assays (Huang et al., 2006). Down-regulation of the *PhSSK1* expression in *Petunia hybrida* had a negative effect on fertility of cross-compatible pollen (Zhao et al., 2010). In *Petunia inflata*, 17 S-locus F-box proteins are assembled into SCF complex with PiSSK1, PiCUL1-P, and PiRBX, indicating that PiSSK1 is responsible for specific function in SI reaction (Li et al., 2016). This hypothesis was confirmed in a *PiSSK1* knockout setting where *PiSSK1* deletion caused incompatibility of mutational pollen in otherwise-compatible pistils. This also revealed the essential role of *SKP1-like* gene in SI reaction (Sun & Kao, 2017). In Rosaceae, many *SKP1-like* gene such as *PavSSK1* from *Prunus avium* (Matsumoto et al., 2012), *PbSSK1* and *PbSSK2* from *Pyrus bretschneideri* and *MdSSK1* from *Malus domestica* (Xu et al., 2013; Yuan et al., 2014) were found to be involved in SI response. In *Citrus*, an *SKP1-like* gene involved in flower development of *pummelo* (*C. grandis*), *CgSKP1*, was identified from ‘Shatian’ cultivar and it was highly expressed in leaf and flower (Chai et al., 2010). In our previous work, we isolated full-length cDNA and DNA sequences of *CrWSKP1* from ‘Wuzishatangju’ (Miao et al., 2015). However, we did not clarify whether these genes are involved in SI response of *Citrus*.

CUL1 protein has been identified from several plant species. In *P. inflata*, PiCUL1-C and PiCUL1-G proteins were obtained and the PiCUL1-G was proposed to assemble into a novel SCF complex with PiSLF and PiSPB1 proteins (Hua & Kao, 2006). SpCUL1 was proposed to be the determinant of interspecies unilateral incompatibility in *Solanum pennellii*. Down-regulation of *SpCUL1* expression impaired the fertility of pollination in wild SI cultivar (Li & Chetelat, 2010; Li & Chetelat, 2014). PiCUL1-P, which is the ortholog of SpCUL1 formed a complex with PiSSK1 and PiRBX1 when a GFP-fused S_2_-SLF1 protein was co-immunoprecipitated with pollen extracts in *P. inflata* (Li et al., 2014). Knockdown of *PhCUL1-P* compromised fertility of cross-compatible pollination in *P. hybrida* (Kubo et al., 2016). Both PavCul1A and PavCul1B protein physically interact with PavSSK1 in *P. avium* (Matsumoto & Tao, 2016; Matsumoto & Tao, 2019). A PbCUL1 protein has been characterized in *P. bretschneideri* (Xu et al., 2013). However, whether *CUL1* gene (s) is involved in SI reaction in *Citrus* remains to be investigated.

The SI reaction in *Citrus* was considered to be S-RNase-based SI type (Zhang et al., 2018; Liang et al., 2020). S-RNase-mediated SI evolved only once before the split of Asteridae (e.g., Solanaceae) and Rosidae (e.g., Rosaceae and Rutaceae). Therefore, S-RNase is proposed to be a putative pistil S-determinant (Igic & Kohn, 2001; Vieira, Fonseca & Vieira, 2008; Zhang et al., 2018). A T2-type *RNase* gene, *CgSL2*, was constitutively expressed and associated with ovary senescence in ‘Zigui shatian’ pummelo (Chai et al., 2011). Another *S-like RNase* gene which shared high sequence identity with *CgSL2* did not show tissue-specific in ‘Wuzishatangju’ (*C. reticulata*) (Miao et al., 2011). In ‘Shatian’ pummelo (*C. grandis*), *CgRNS3* possessed several common characteristics of the pistil determinant of SI and was specifically expressed in pistil (Liang et al., 2017), however, it was not anchored into the S-locus (Liang et al., 2020). Pistil-specific *CtRNS3* from *Citrus tamurana* showed S_1_-genotype-dependent in different cultivars, implying that *CtRNS3* may serve as the *S*_1__-_gene (Honsho et al., 2019). Liang et al. (2020) studied the S-locus in pummelo and concluded that *S-RNase* and *SLF* mediate SI reaction in *Citrus*.

‘Wuzishatangju’ (*C. reticulata* Blanco) is a natural mutant. Cytological studies show that gametophytic SI causes seedlessness in ‘Wuzishatangju’ by inhibiting fertilization in the ovary (Ye et al., 2009). Several genes related to SI of ‘Wuzishatangju’ have been obtained by suppression-subtractive hybridization (SSH) cDNA library and RNA-Seq technology. However, it is still not known which factor regulates SI reaction in ‘Wuzishatangju’ (Miao et al., 2013; Ma et al., 2017). In this study, 16 pollen-specific *F-box* genes (*CrFBX1-CrFBX16*), one *SKP1-like* gene (*CrSKP1-e*) and two *CUL1* genes (*CrCUL1A* and *CrCUL1B*) were cloned from ‘Wuzishatangju’ by genome-wide analyses. Among *CrFBX* genes, 13 *CrFBXs* (*CrFBX1-CrFBX12* and *CrFBX14*) were homologous with *SFBB* and *SLFL*, 10 *CrFBXs* (*CrFBX1*-*CrFBX10*) were located into S-locus. *CrSKP1-e* and *CrCUL1A* were predominately found in pollen of ‘Wuzishatangju’ while *CrCUL1B* was least abundant in pollen. Yeast two-hybrid, in vitro binding and LCI assays showed that CrSKP1-e links with a subset of CrFBX proteins and binds to both CrCUL1A and CrCUL1B. These results suggested that CrSKP1-e acts as an adaptor in the assembly of SCF-type E3 ligase in ‘Wuzishatangju’.

## Materials and Methods

### Plant materials

‘Wuzishatangju’ (self-incompatible) (*C. reticulata*) and ‘Chuntianju’ (self-compatible) (*C. reticulata*) mandarins were planted in an orchard at South China Agricultural University (Guangzhou, China). Young leaves, petals, filaments, stigmas, styles and ovaries were collected, immediately frozen in liquid nitrogen and stored at −80 °C for later use. Buds were collected one day before anthesis and anthers were dried in an oven at 28 °C for 48 h. Pollen grains were gathered by filtering with 75 µm stainless sieve after desiccation and then stored at −80 °C. ‘Chuntianju’ pollen was used to clone *CrFBX7*.

### Identification of F-box, SKP1-like and CUL1 proteins

F-box, SKP1-like and Cullin (CUL) family proteins were retrieved using in silico method based on the Hidden Markov Model (HMM) profile of F-box domain seed (PF00646), SKP1 seed (PF01466) and CUL seed (PF00888), respectively, obtained from the Pfam database (http://pfam.xfam.org/) as a query to search the predicted *C. clementina* proteome (https://phytozome.jgi.doe.gov/pz/portal.html) using HMMER software package 3.0 (http://hmmer.org/download.html) with E ≤1, (Finn, Jody & Eddy, 2011). The MAFFT program was used for multiple sequence alignments based on the amino acid sequences, while the MEGA software (Version 7.0) with 1,000 replicated bootstrap tests were used for phylogenetic analyses (Kumar, Stecher & Tamura, 2016; Yamada, Tomii & Katoh, 2016).

### Expression analyses of candidate genes

Total RNA was isolated and digested with DNase I using Plant RNA Kit (Huayueyang, Beijing, China). Total RNA (0.4–0.5 µg) was used to synthesize the first-strand cDNA with oligo (dT) primer according to the manufacturer’s instructions using a RevertAid First Strand cDNA Synthesis Kit (ThermoFisher, USA). The expression patterns of *CrFBXs*, Cr*SKP1-e*, *CrCUL1A* and *CrCUL1B* were analyzed with quantitative real-time PCR (qRT-PCR) and normalized by the expression level of *Actin* gene (*Actin_F*: CATCCCTCAGCACCTTCC and *Actin_R*: CCAACCTTAGCACTTCTCC) (Zhou et al., 2010). qRT-PCR was conducted in ABI 7500 real-time PCR System (Applied Biosystems, CA, USA) using the SYBR qPCR Mix (Vazyme, Nanjing, China). The 20 µL reaction mixture contained about 1 µL template cDNA, 0.2 µM of each forward and reverse gene-specific primers and 10 µL SYBR. The relative expression levels were calculated using the 2^−ΔΔ*C*^T formula (Livak & Schmittgen, 2001). For qRT-PCR, samples were collected during three consecutive days. Samples from each day served as a biological repeat.

The full-length cDNA of pollen-specific *CrFBXs*, *CrSKP1-e*, *CrCUL1A* and *CrCUL1B* were cloned from ‘Wuzishatangju’ using gene-specific primer pairs based on *C. clementina* genome reference; transcriptome datasets (https://figshare.com/articles/Citrus_reticulata_Blanco-Unigene_5-3_fa/12198627) (Ma et al., 2017) and resequencing data (https://figshare.com/articles/wuzi_rmdup_bam/11880303). C*rFBX7* was cloned from ‘Chuntianju’ pollen. Fragments were purified and cloned into the pEASY-Blunt vector (Transgen, Beijing, China) for sequencing. All the primer pairs are presented in Table S1.

### Yeast two-hybrid (Y2H) analysis

Full-length CDS of *CrFBXx* (*x* represents the number of *CrFBX*, the full-length CDS of *CrFBX7* was cloned from ‘Chuntianju’) was cloned into pGBKT7 vector (Clontech, USA), which was respectively digested by *Eco* R I and *Bam* H I enzymes, to express fusion proteins with GAL4 binding domain (BD). *CrSKP1-e* was cloned into the pGADT7 vector (Clontech, USA) to produce fusion proteins with the GAL4 activation domain (AD). Different combinations with BD and AD vectors were co-transformed into Y2HGold strain (Clontech, USA) and incubated with SD/-Leu/-Trp at 30 °C for 4 d. Three clones were respectively dotted on SD/-Leu/-Trp/-Ade/-His medium containing 200 ng/mL Aureobasidin A (AbA) (TaKaRa, Japan) and 40 µg/mL X- α-gal (TaKaRa, Japan) and then cultivated at 30 °C for 5 d.

### Luciferase complementation imaging (LCI) assay

CrCUL1A and CrCUL1B proteins were truncated at the N-terminal region (CrCUL1A-N, 1-415; CrCUL1B-N, 1-415, respectively). All fragments were cloned into pCAMBIA-nLuc vector and *CrSKP1-e* was cloned into a pCAMBIA-cLuc vector (Chen et al., 2008). The constructed plasmids were individually transformed into *Agrobacterium* GV3101 strains and incubated at 28 °C for 2-3 d on the YEP (yeast extract peptone) medium supplemented with 25 µg/mL rifampicin (Rif) and 50 µg/mL kanamycin (Kan). A single colony was incubated at 200 rpm and 28 °C for 8-10 h in YEP liquid medium containing 25 mg/L Rif and 50 mg/L Kan. The presence of the corresponding plasmid was verified by PCR. Then 30 µL of positive *Agrobacterium* suspension was inoculated in 15 mL YEP liquid medium and incubated until OD_600_ reached 0.6–0.8. After centrifugation for 5 min at 6,000 rpm and 25 °C, the medium was discarded. The pellet was resuspended and the OD_600_ adjusted to 0.2 with MAA buffer (10 mM MES, 10 mM MgCl_2_, 0.1 mM acetosyringone, pH5.6) for infiltration of 3–4 weeks old *Nicotiana benthamiana* (16-h day/8-h night, 25 °C). For the co-infiltration assay, the suspension was mixed with an equal volume of *Agrobacterium* strains containing the recombinant plasmid. After 3 d, the Luciferase Assay Substrate (Promega, USA) was infiltrated into the leaf and the reaction imaging was captured by a low-light cooled charge-coupled device (CCD) imaging system (Bio-Rad, USA). For luciferase activity, 0.05 g sample was ground in liquid nitrogen and 500 µL tissue lysis reagent (Promega, USA) was added and centrifuged at 4 °C. The luminescence signal was detected using the chemiluminescence analysis system (Thermo Scientific, USA) after mixing 20 µL supernatant with 100 µL Luciferase Assay Substrate (Promega, USA). All assays were replicated thrice.

### In vitro binding assay

For CrSKP1-e and CrFBX proteins binding assay, the full-length of *CrFBX2* and *CrFBX7* (derived from ‘Chuntianju’) and *CrFBX9*, *CrFBX13* and *CrFBX15* were fused with MBP (maltose-binding protein) tag and cloned into pET-28a vector. *CrSKP1-e* was cloned into pGEX-4T-2. All the constructs and negative control (pET28a-MBP and pGEX-4T-2) were transformed into BL21 (DE3) strains to express MBP, MBP-CrFBX2, MBP-CrFBX7, MBP-CrFBX9, MBP-CrFBX13, MBP-CrFBX15, GST (glutathione S-transferase) and GST-CrSKP1-e proteins. For CrSKP1-e and CrCUL1 binding assay, the full length of *CrSKP1-e* was cloned into pET28b and the N-terminal regions of CrCUL1A (1-415) and CrCUL1B (1-415) proteins were respectively cloned into pGEX-4T-2. All the constructs and negative control (pGEX-4T-2) were transformed into Rosetta (DE3) to express His-CrSKP1-e, GST-CrCUL1A-N and GST-CrCUL1B-N proteins. For protein accumulation, three clones were incubated in the LB (Lysogeny broth) medium containing 100 mg/L ampicillin at 37 °C for 6 h. The culture was then diluted (about 1:100) into a fresh LB medium for further incubation until the OD_600_ was 0.6–0.8. Approximately 0.2 mM isopropyl- β-D-thiogalactosidase (final concentration) was added into the culture and incubated at 28 °C under shaking condition (200 rpm) for 5–6 h to induce the recombinant protein accumulation. For His-CrSKP1-e protein purification, the bacteria culture was sonicated and purified with Ni-NTA His Bind resin (TransGen, China) according to the manufacturer’s instructions and then exchanged with PBS (phosphate-buffered saline) buffer (140 mM NaCl, 2.7 mM KCl, 10 mM Na_2_HPO_4_, 1.8 mM KH_2_PO_4_, pH7.4–7.5) using an Amicon Ultra-15 (Millipore, 10K) The MBP fused proteins were sonicated and purified using Dextrin Beads (SMART-lifesciences, Changzhou, China) according to the manufacturer’s instructions. Protein concentration was measured using Bradford Protein Assay Kit (TaKaRa, Japan).

For binding assays, bacteria suspension expressing GST and GST fusion proteins were sonicated in PBS buffer and the supernatant was reacted with 40 µL of 50% slurry of Glutathione Sepharose 4B (GE Healthcare, USA). The mixture was incubated at 4 °C under soft shaking condition for 2 h. About 30 µg His-CrSKP5, MBP, MBP-CrFBX2, MBP-CrFBX7, MBP-CrFBX9, MBP-CrFBX13 and MBP-CrFBX15 were respectively incubated with protein-bound Glutathione Sepharose 4B at 4 °C under soft shaking conditions overnight. The beads were washed five times with 10 × PBS buffer. The protein-bound beads were boiled and separated with SDS-PAGE (sodium dodecyl sulfate-polyacrylamide gel electrophoresis). The His-CrSKP1-e was detected with an anti-His monoclonal antibody and the MBP-fused proteins were detected with an anti-MBP polyclonal antibody (Yeasen, China).

## Results

### Identification of pollen-specific *F-box* genes

**Figure 1:**
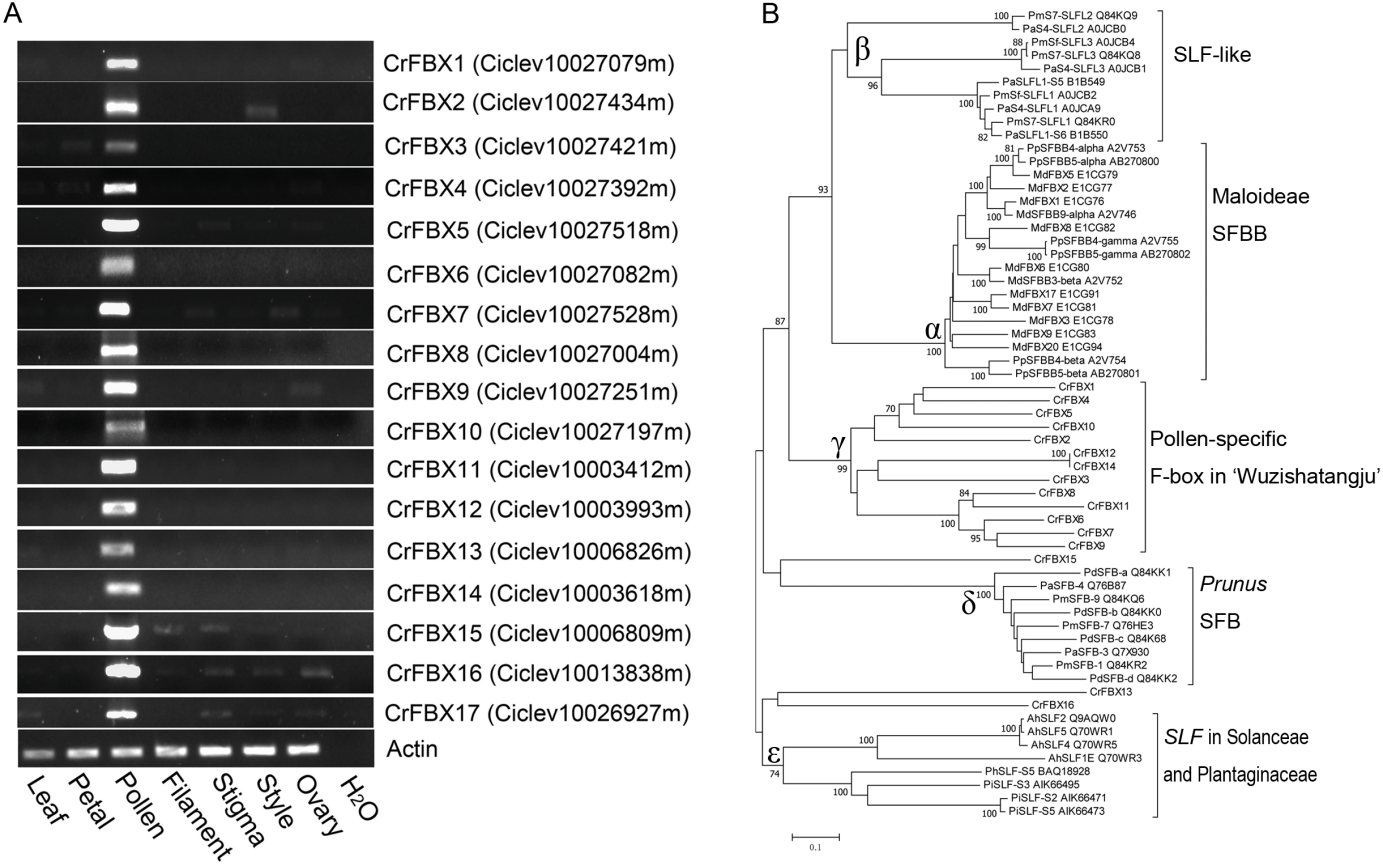
Expression analyses and phylogenetic relationships of *CrFBX* genes from ‘Wuzishatangju’. (A) PCR analyses of the expression pattern of the 17 *CrFBX* genes, using the expression of the *actin* gene as the reference. Expression was assessed in the leaf, petal, pollen, filament, style and ovary. (B) Phylogenetic relationships between CrFBX and SLF/SFBB/SLFL/SFB proteins. The numbers of each interior branch are the percentage of bootstrap values. The numbers after each SLF/SFBB/SLFL/SFB proteins are the UniProt accession numbers. Md-, *Malus* ×*domestica*; Pm-, *Prunus mume*; Pd-, *Prunus dulcis*; Pa-, *Prunus avium*; Cr-, *Citrus reticulata*; Pi-, *Petunia inflata*; Ah-, *Antirrhinum hispanicum*.

An HMMER search was conducted for genome-wide investigation of the number and domain organization of the F-box proteins in *C. clementina* (v1.0). A total of 298 non-redundant F-box proteins and 46 F-box associated (FBA) subfamily F-box proteins were identified (Fig. S1). *SLFs* and *SLFLs* genes are significantly expressed in mature pollen and contain a typical FBA domain in the C-terminal region. Therefore, semi-quantitative RT-PCR was performed to investigate the tissue-specific expression of the 46 FBA subfamily genes in different tissues (leaf, petal, filament, pollen, stigma, style and ovary) of ‘Wuzishatangju’ (Fig. 1A and Figs. S2A, S2B). In total, 17 *F-box* genes (termed *CrFBX1-CrFBX17*) were significantly expressed in pollen. Full-length coding sequences of all the *CrFBX* genes except *CrFBX17* (Ciclev10026927m) were cloned from the pollen cDNA pool of ‘Wuzishatangju’ and aligned with homologous transcripts of *C. clementina*. The identity ranged from 80% to 100% (Table S2 ). However, a 1-base pair (bp) deletion in the 3′-terminus of *CrFBX7* in ‘Wuzishatangju’ was found (Fig. S3). To verify the *CrFBX7* gene in *C. reticulata*, the full-length CDS sequence of *CrFBX7* from self-compatible ‘Chuntianju’ (*C. reticulata*) was obtained and used for further analyses (Fig. S3).

The phylogenetic relationships of pollen-specific F-box proteins with SFBBs (SFBs), SLFs and SLFLs, derived from Rosaceae, Solanaceae and Plantaginaceae were explored. Full-length amino acid sequences of pollen-specific CrFBX proteins, SLFs from *Petunia* and *Antirrhinum*, SFBs and SLFLs from *Prunus* and SFBBs from Maloideae were aligned using the MAFFT program and a phylogenetic tree was constructed (Fig. 1B). The topological structure included clades α (SFBB), β (SLFL), γ (CrFBX), δ (SFB) and ε (SLF). All clades showed stronger bootstrap values (≥92%). The pollen factor (SFB) from *Prunus* failed to cluster with SFBB, SLFL and SLF clades but formed a single cluster. This was consistent with the finding that SFB and SFBB/SLFL/SLF originated from distinct ancestral genes, whereas the functions of SFBB and SLFL in the S-RNase-based SI system in flowering plant derived from a common origin (Akagi et al., 2016). Besides, 13 pollen-specific CrFBX proteins were clustered into the γ clade with a bootstrap value of 99%, which were different from those of SFBB, SLFL, SFB and SLF (Fig. 1B). CrFBX13, CrFBX16 and CrFBX15 were not homologous with the S-locus F-box proteins. To characterize *SLF* genes in ‘Wuzishatangju’, *CrFBX1*-*CrFBX16* were mapped into the *C. clementina* genome. The results showed that 10 *CrFBX* (*CrFBX1*-*CrFBX10*) was located in the S-locus (Fig. S4) (Liang et al., 2020). These results suggested that the S-locus, which is similar to the apple or pummelo, is also existent in the ‘Wuzishatangju’ genome.

### Pollen-specific expression analyses of *SKP1-like* genes

**Figure 2:**
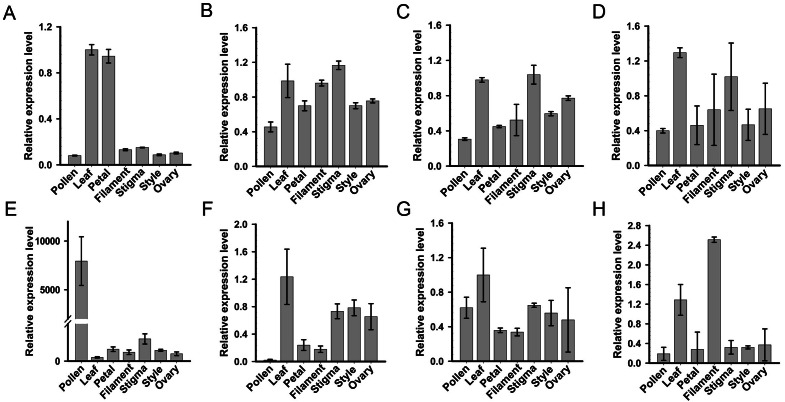
Expression analyses of *SKP1* family genes in different tissues. Quantitative RT-PCR analysis of the eight *SKP1* family genes, using the Hidden Markov Model (HMM) profile of the SKP1 family protein as a query. (A) *CrSKP1-a*; (B) *CrSKP1-b*; (C) *CrSKP1-c*; (D) *CrSKP1-d*; (E) *CrSKP1-e*; (F) *CrSKP1-f*; (G) *CrSKP1-g* and (H) *CrSKP1-h*.

For systematic identification of the SKP1 family protein(s), which potentially serve as adaptors for assembling the SCF complex in *C. reticulata*, we conducted an HMMER search for available pollen and pistil transcriptome datasets of ‘Wuzishatangju’ (Ma et al., 2017) using the Hidden Markov Model (HMM) profile of the SKP1 family protein as a query. Eight independent genes (termed *CrSKP1-a* to *CrSKP1-h*) were characterized (Fig. S5B). Expression profiles of all the *SKP1* family genes in *C. reticulata* were analyzed with qRT-PCR (Fig. 2). Among these *SKP1* candidate genes, only *CrSKP1-e* showed higher pollen-specific expression patterns in ‘Wuzishatangju’ (Fig. 2E). In addition, while *CrSKP1-f* was less expressed in pollen (Fig. 2F), *CrSKP1-a* was highly expressed in the leaf and the petal (Fig. 2A). *CrSKP1-h* was also highly expressed in the leaf and the filament (Fig. 2H). However, *CrSKP1-b*, *CrSKP1-c*, *CrSKP1-d* and *CrSKP1-g* showed constitutive expression patterns in all ‘Wuzishatangju’ tissues (Figs. 2B,  2C,  2D,  2G).

The HMM profile of SKP1 was used to search the *C. clementina* genome for the comprehensive identification of the *SKP1* genes. A total of 13 non-redundant *SKP1* genes were characterized (Figs. S5A, S5B). For phylogenetic analyses, an unrooted Neighbor-Joining (NJ) tree was constructed based on the multiple sequence alignments. Three groups (group I, group II and group III with 88%, 89% and 100% bootstrap values, respectively) were distinctly clustered (Fig. S5A). The transcripts and expression patterns of *SKP1* genes were investigated in *C. reticulata*. One gene was not detected in pollen (Fig. S5C) and three genes were an unbiased expression in *C. reticulata* pollen (Figs. S5D–S5F). The results from expression analyses showed that the *CrSKP1-e* gene was the candidate gene involved in the SI reaction in ‘Wuzishatangju’.

**Figure 3:**
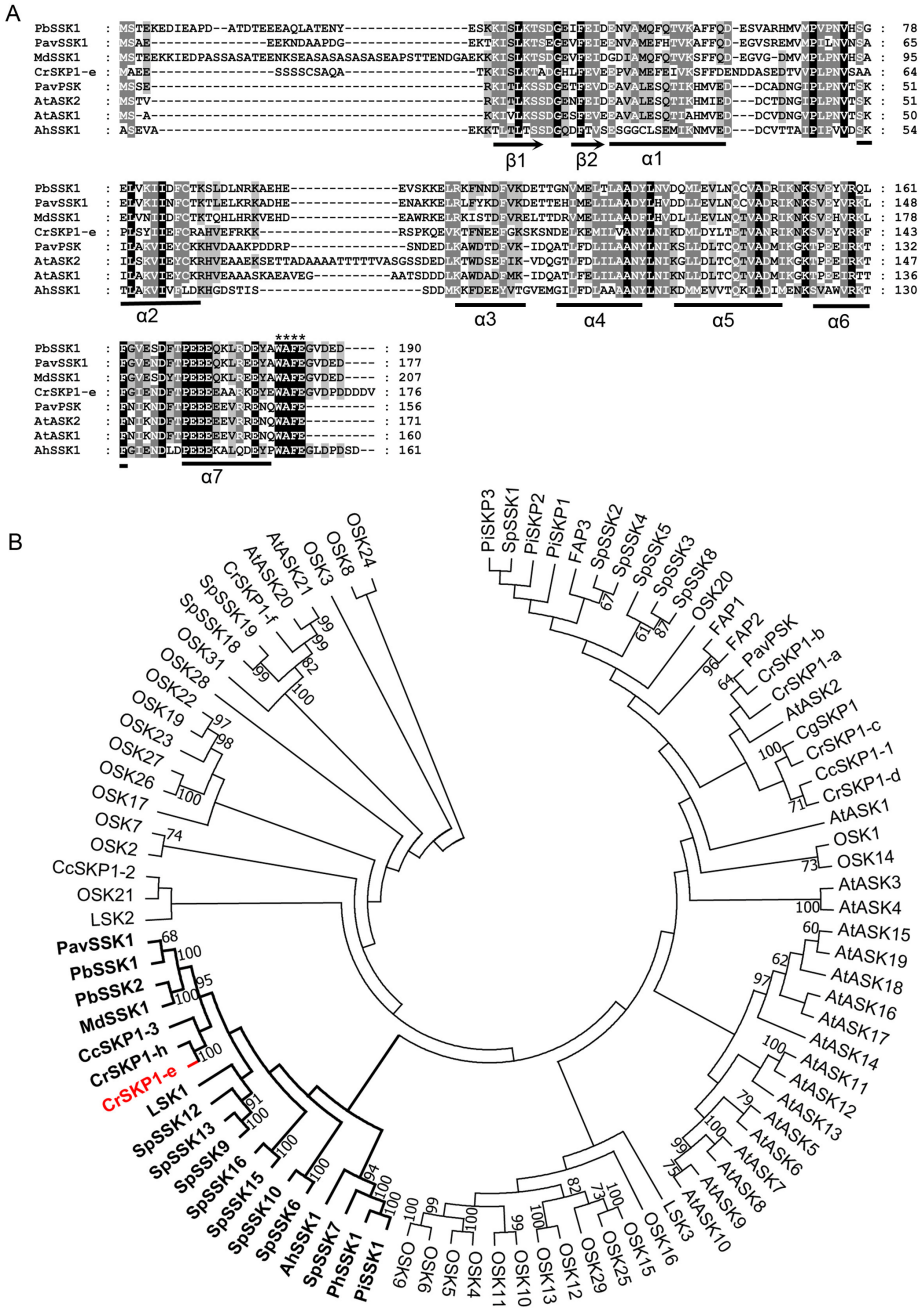
Multiple sequence alignment and phylogenetic analyses of pollen-specific CrSKP1-e. (A) CrSKP1-e protein consisted of β1 and β2 sheets in the N-terminus, which connects the F-box domain and the seven helices (α1- α7) binding the CUL1 protein in the C-terminus. (B) An unrooted neighbor-joining (NJ) tree of CrSKP1-e and 84 SKP1-like proteins in other plants. The CrSKP1-e protein was well clustered with the SSK1 protein involved in S-RNase-based SI reaction in Rosaceae, *Antirrhinum* and Solanaceae, but was not the orthologs from rice (OSK1-OSK31) and *Arabidopsis* (AtSK1-AtSK21). All the accession numbers are presented in Table S3.

### Cloning and phylogenetic analyses of *CrSKP1-e*

The full-length coding sequence of pollen-specific *CrSKP1-e* was cloned from ‘Wuzishatangju’. *CrSKP1-e* encoded 176 residues and was identical with the reference transcript of Ciclev10006034m in *C. clementina* (Figs. 3A, Figs. S5B). However, *CrSKP1-e* was different from *CrWSKP1* obtained from the suppression subtractive hybridization (SSH) library of ‘Wuzishatangju’ (Miao et al., 2015). The CrSKP1-e protein consisted of β1 and β2 sheets in the N-terminus, which connects the F-box domain and the seven helices (α1- α7) binding the CUL1 protein in the C-terminus (Fig. 3A). Compared with a typical SKP1 protein, the CrSKP1-e protein not only contained the conserved terminal residues ‘WAFE’ found in most SKP1 family proteins, but also had the unique C-terminal tail ‘GVDPDDDDV’ (Fig. 3A). The unique residue tail following the ‘WAFE’ domain was characterized in the SKP1-like protein, which was exclusively involved in S-RNase-based the SI reaction in Rosaceae (e.g., PbSSK1, PavSSK1 and MdSSK1) and *Antirrhinum* (e.g., AhSSK1) (Fig. 3A). Phylogenetic analyses were conducted based on the amino acid residues of the 84 SKP1-like proteins (Table S3). The CrSKP1-e protein was well clustered with the SSK1 protein involved in the S-RNase-based SI reaction in Rosaceae, *Antirrhinum* and Solanaceae (Fig. 3B). Furthermore, the CrSKP1-e protein was not the orthologs from rice (OSK1-OSK31) and *Arabidopsis* (AtSK1-AtSK21) (Fig. 3B). A relatively lower expression level of *CrSKP1-h* was detected in the pollen, although it was homologous with *CrSKP1-e* (Fig. 2H). This indicated that *CrSKP1-h* might not be a candidate gene involved in the SI reaction in ‘Wuzishatangju’. These results suggested that *CrSKP1-e* potentially serves as a link for assembling the SCF-type E3 complex involved in the SI response of ‘Wuzishatangju’.

### Identification and cloning of *CUL1* genes

**Figure 4:**
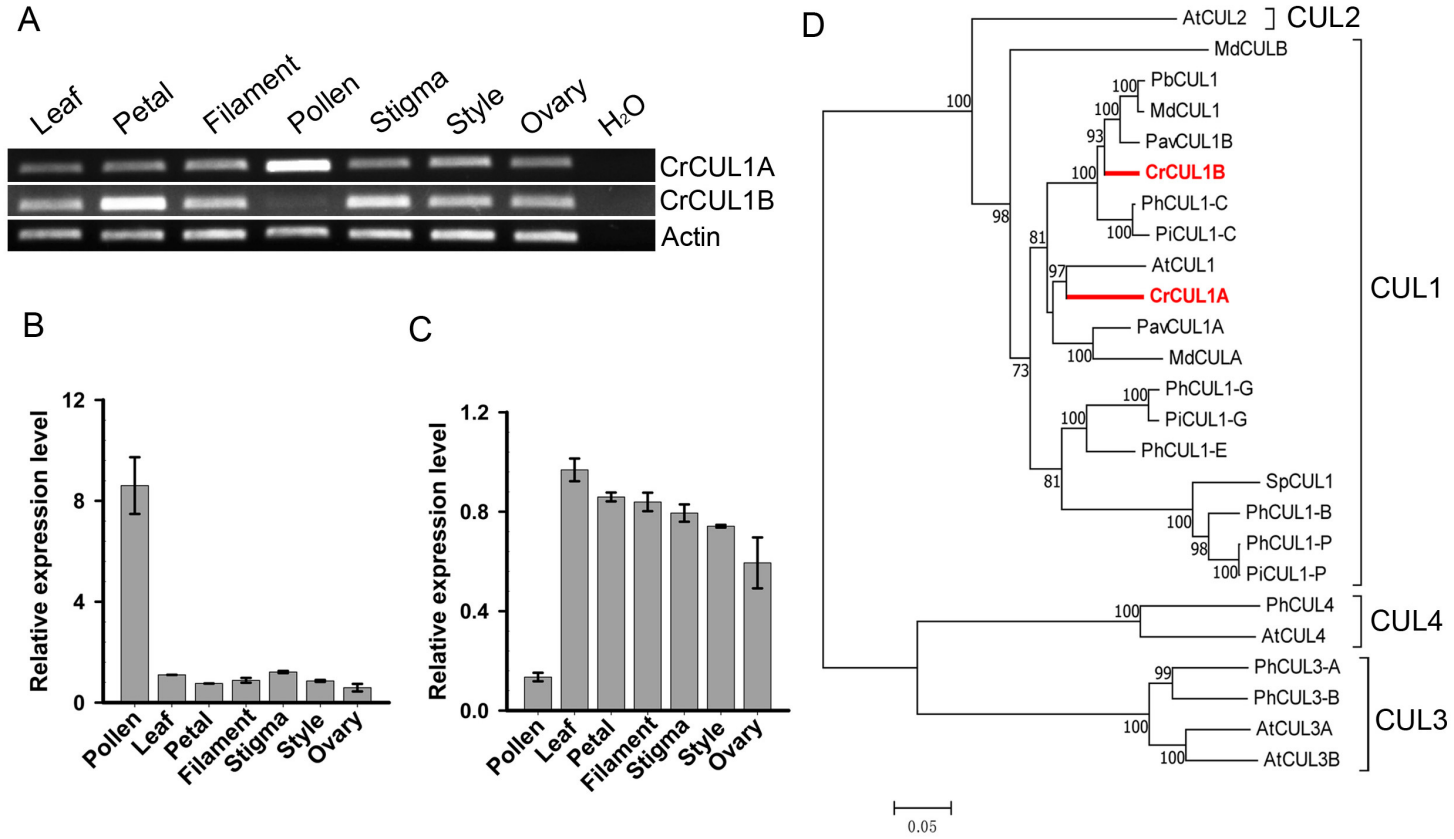
Expression analyses and phylogenetic relationships of *CrCUL1A* and *CrCUL1B* in *C. reticulata*. (A) Semi-quantitative PCR analysis of the expression patterns of *CrCUL1A* and *CrCUL1B* genes, using the expression of the *actin* gene as the reference. Quantitative RT-PCR analyses of (B) *CrCUL1A* and (C) *CrCUL1B*. (D) Unrooted NJ tree of the deduced Cullin (CUL) proteins constructed using MAGA7 software. The numbers of each interior branch are the percentage of bootstrap values. At-, *A. thaliana*; Md-, *M. domestica*; Pb-, *P. bretschneideri*; Pav-, *P. avium*; Ph-, *P. hybrida*; Pi-, *P. infilata*; Cr-, *C. reticulata*; Sp-, *S. pennellii*. The GenBank accession numbers are presented in Table S3.

To characterize the CUL1 (Cullin1) protein(s) that potentially serve as scaffolds for the assembly of the SCF complex in ‘Wuzishatangju’, a genome-wide analysis of the CUL family sequences in *C. clementina* was conducted. Sixteen CUL-related proteins were identified (Table S4). To investigate the CUL1 homologs, an NJ tree was generated to assess the phylogenetic relationships between the candidate CUL proteins and the known AtCUL1-AtCUL4 proteins from *A. thaliana*. Three genes (Ciclev10018125m, Ciclev10019010m and Ciclev10004406m) belonging to the AtCUL1 subgroup were obtained (Fig. S6). No expression of Ciclev10018125m was detected in all the tissues tested (data not shown). The full-length coding sequences of the two CUL1 genes termed *CrCUL1A* and *CrCUL1B* were cloned from ‘Wuzishatangju’. Compared with *C. clementina*, the coding sequence of *CrCUL1A* and Ciclev10019010m from ‘Wuzishatangju’ shared identical sequence while the two single-nucleotide polymorphisms (SNP) sites were detected between *CrCUL1B* and Ciclev10004406m (Figs. S7A, S7B). *CrCUL1A* showed preferential expression in mature pollen compared to the other tissues (Figs. 4A, 4B). On the contrary, *CrCUL1B* showed unbiased expression in all tissues except for the lower expression level detected in pollen (Figs. 4A, 4C). Phylogenetic analyses indicated that *CrCUL1A* and *CrCUL1B* belonged to Rosaceae-related CUL1 groups. However, *CrCUL1A* and *CrCUL1B* failed to cluster with *PiCUL1-P*, which is considered as an essential component of the SCF complex for the non-self-recognition system in *Petunia* (Fig. 4D)*.* These results suggested that CrCUL1A and CrCUL1B potentially interact independently or jointly with the non-S-locus ortholog, CrSKP1-e, and form an SCF complex involved in the SI reaction in *C. reticulata.*

### Interaction of CrSKP1-e with CrFBX proteins using Y2H and in vitro binding assays

**Figure 5:**
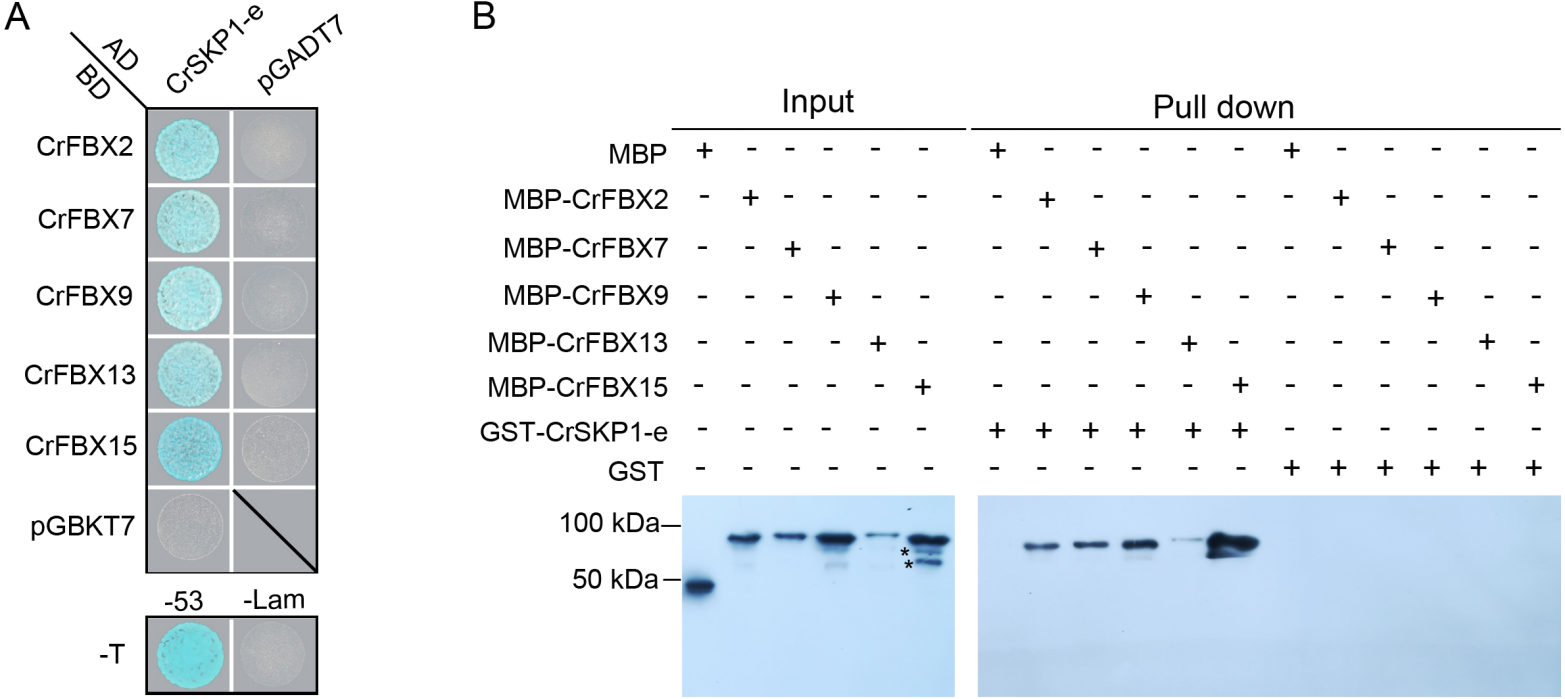
Interaction of CrSKP1-e and CrFBX proteins. (A) The interaction of CrSKP1-e and CrFBX proteins was evaluated using the Y2H assay. A combination of BD-CrFBX*x* (*x* means the number of CrFBX proteins) vectors and AD-CrSKP1-e vectors were co-transformed into Y2HGold strain and selected on SD/-L/-T medium (Fig. S8). The positive transformants were inoculated on a selective medium SD/-A/-H/-L/-T containing X- α-Gal and AbA for growth. pGADT7-T and pGBKT7-Lam were used as negative controls. pGADT7-T and pGBKT7-53 were used as positive controls. (B) The interaction of CrSKP1-e and CrFBX proteins was evaluated using the in vitro binding assay. GST (negative control) and GST-CrSKP1-e were reacted with Glutathione Sepharose 4B, and then GST-bound beads were incubated with MBP (negative control) and MBP-fused proteins. The MBP signal was detected using western blotting. A asterisk (*) indicates the non-specific proteins.

The interactions of pollen-specific CrFBX1-CrFBX16 with CrSKP1-e were investigated using the Y2H and in vitro binding assays. The growth of Y2HGold strains showed that the CrSKP1-e protein could interact with CrFBX2, CrFBX7, CrFBX9, CrFBX13 and CrFBX15 proteins (Fig. 5A). However, based on the phylogenetic analyses and genomic physical location results, *CrFBX13* and *CrFBX15* were not the S-locus genes. GST (negative control) and GST-CrSKP1-e were reacted with Glutathione Sepharose 4B. The GST-bound beads were then incubated with MBP (negative control) and MBP-fused proteins. The results showed that CrFBX2, CrFBX7, CrFBX9, CrFBX13 and CrFBX15 were bound to CrSKP1-e (Fig. 5B). Taken together, these results suggested that CrSKP1-e protein could interact with a cluster of F-box proteins, including some non-S-locus F-box proteins.

### Interaction of CrSKP1-e with CrCUL1A and CrCUL1B proteins

**Figure 6:**
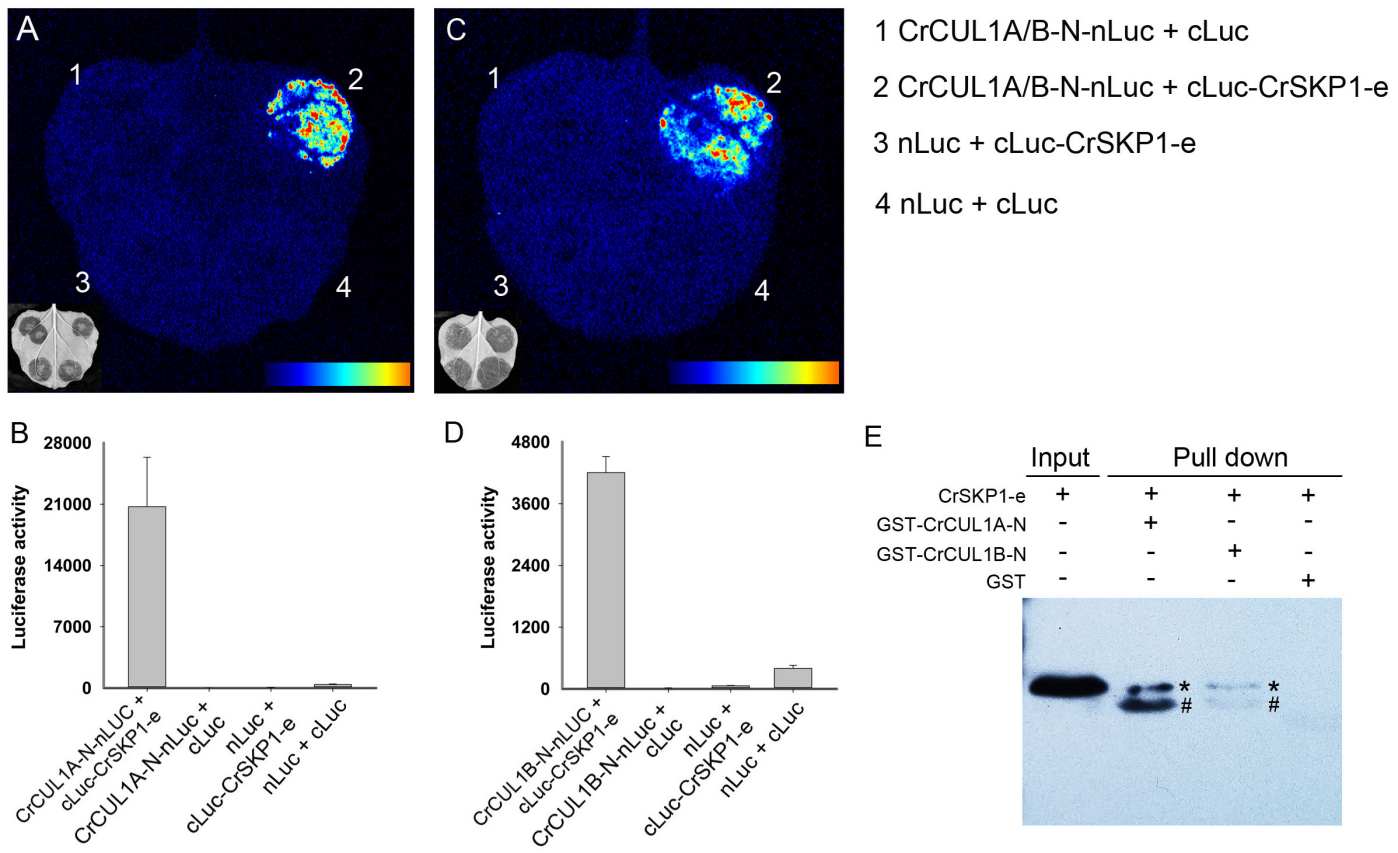
Interaction assay of CrSKP1-e and CrCUL1 proteins. Luciferase imaging (A) and luciferase activity (B) during co-expression of CrSKP1-e and the N-terminal region of CrCUL1A (CrCUL1A-N). Luciferase imaging (C) and luciferase activity (D) during co-expression of CrSKP1-e with the N-terminal region of CrCUL1B (CrCUL1B-N). (E) *In vitro* binding assay for CrSKP1-e and CrCUL1 proteins. As asterisk (*) indicated CrSKP1-e protein, a number sign (#) indicated non-specific proteins.

To examine the potential interaction between CrSKP1-e and CUL1 proteins, the LCI assay was conducted. As shown in Fig. 6, co-expression of CrSKP1-e (cLuc-CrSKP1-e) and the N-terminal regions of CrCUL1A (CrCUL1A-N-nLuc) in *N. benthamiana* leaves resulted in strong luciferase activities (Figs. 6A, 6B). Similarly, luciferase activity was also strongly detected during the co-expression of CrSKP1-e (cLuc-CrSKP1-e) and the N-terminal regions of CrCUL1B (CrCUL1B-N-nLuc) (Figs. 6C, 6D). In vitro binding assay indicated that His-CrSKP1-e reacted with GST-CrCUL1A-N and GST-CrCUL1B-N (Fig. 6E). These results suggested that the CrSKP1-e protein could interact with the N-terminal regions of CrCUL1A and CrCUL1B protein.

## Discussion

### Identification of the S-locus *F-box* genes in *C. reticulata*

The F-box family proteins are identified by the existence of the 60 conserved residue regions in the N-terminal region called the F-box domain, which acts as a receptor to bind to the SKP subunit (Qiao et al., 2004; Williams et al., 2014; Zhao et al., 2002). The number of *F-box* genes vary widely between species. In plants, at least 692, 337, 779 and 285 *F-box* genes have been characterized in *Arabidopsis*, poplar, rice and chickpea, respectively (Xu et al., 2009; Gupta et al., 2015). In this study, 298 non-redundant F-box superfamily proteins were obtained from *C. clementina*, which represents about 1.2% of the annotated proteins in the *C. clementina* (v1.0) genome. In addition to the F-box domain, several F-box proteins such as Kelch, Leucine-rich repeat, FBD, PP2 motif and FBA domains, were predicted to contain a C-terminal region. This indicates that the F-box proteins are responsible for the significant function during growth and development, disease resistance and reproduction (Fig. S1) (Gagne et al., 2002). Most of the F-box proteins contain the C-terminal protein-protein interaction domains. In clementine mandarin, the most abundant F-box type is the FBA subfamily (46 proteins). Some members of the FBA subfamily act as pollen determinants to recognize S-RNase or as ‘inhibitors’ (SLFLs from *Prunus*) to inactivate self-S-RNase during S-RNase-based SI response in Rosaceae, Solanaceae and Plantaginaceae. In *Citrus*, information about the genetic linkage between SI and the S-genotype has been elucidated in pummelo (Liang et al., 2020). SI reaction in mandarin is suggested to be an S-RNase-mediated system (Liang et al., 2020). In the present study, 16 *F-box* genes derived from the FBA subfamily were obtained based on the *C. clementina* genome and ‘Wuzishatangju’ pollen transcript data. These were specifically expressed in the ‘Wuzishatangju’ pollen. Based on the phylogenetic analyses, not all the pollen-specific *F-box* genes were orthologous to *SFB* genes in *Prunus*; and 13 genes were clustered with *SFBB*/*SLFL* (Fig. 1B). Physical organization analysis suggested that *CrFBX1*-*CrFBX10* genes were located in the S-locus in *Citrus*, indicating that intrahaplotypic diversity of *CrFBX* potentially causes the polymorphism of pollen determinants involved in S-RNase recognition. Furthermore, *CrFBX7* had a 1-bp deletion at the 3′-terminus in ‘Wuzishatangju’, which is predicted to trigger translation termination failure. However, it is not clear whether non-functional *CrFBX7* is involved in the SI reaction of ‘Wuzishatangju’.

### Identification of *SKP1-like* and *CUL1* genes

The SKP1 protein acts as an adaptor to bind the F-box and CUL1 protein for assembling of the canonical SCF-type E3 ligase complex, which mediates the loading of ubiquitin to various substrates. In *Arabidopsis*, SKP1-like protein 13 regulates seed germination and seedling growth (Rao et al., 2018). Pollen-specific SKP1-like proteins are essential for pollen tube elongation in lily (Chang et al., 2009). SKP1 is also involved in salt and drought tolerance in soybean (Chen et al., 2018b). In this study, the genome-wide identification of *SKP1* family genes was conducted to identify candidate non-S factors based on the *C. clementina* genome, which are potentially involved in the SI reaction in ‘Wuzishatangju’. The results from the expression analyses indicated that the *CrSKP1-e* gene was significantly up-regulated in pollen compared to the other tissues of ‘Wuzishatangju’. The expression pattern of *CrSKP1-e* in ‘Wuzishatangju’ was consistent with that of *MdSSK1* in *M. domestica*, *PbSSK1* and *PbSSK2* in *P. bretschneideri*, *PhSSK1* in *P. hybrida* and *PavSSK1* in *P. avium* (Zhao et al., 2010; Matsumoto et al., 2012; Xu et al., 2013; Yuan et al., 2014). These results suggest that *CrSKP1-e* genes are mainly responsible for pollen function. In addition, the CrSKP1-e protein had a conserved C-terminal region, a motif ‘GVDPDDDDV’ following the conventional ‘WAFE’ motif, which is found in most SKP1 family proteins. Interestingly, this unique tail in the C-terminal region of the *SKP1-like* genes involved in the S-RNase-based SI reaction is always present. For instance, this tail is ‘GVDED’ in Rosaceae. However, it is not invariable in Solanaceae and Plantaginaceae but for the ‘D’ in the last position, which is conserved (Aguiar et al., 2015). Phylogenetic relationships suggested that *CrSKP1-e* clustered with *PavSSK1*, *MdSSK1*, *PbSSK1* and *PbSSK2*. These results imply that *CrSKP1-e*, homologous to *SSK1*, is also present in ‘Wuzishatangju’.

Different CUL1 homologs, such as PiCUL1-G and PiCUL1-C proteins in *P. inflata*, Cullin1-like protein in *Antirrhinum* and CUL1 orthologs in Rosaceae, were proposed to be one of the members of the SCF complex in the SI reaction (Hua & Kao, 2006; Qiao et al., 2004; Xu et al., 2013). In *P. inflata*, though PiCUL1-G, SBP1 and SLF were known to be the novel E3 ligase complex mediating S-RNase ubiquitination, PiCUL-P, another CUL1 homolog protein, was proved to be a component of the canonical SCF ligase complex involved in non-self-recognition in the SI reaction (Kubo et al., 2016). In *P. avium*, PavCUL1A and PavCUL1B were considered to be components of a functional SCF^SFB^ complex (Matsumoto et al., 2012). In this study, the Cullin family proteins were first identified from *C. clementina*. The three CUL proteins were closely related to the AtCUL1 protein (Fig. S6). Only the CUL1 subfamily proteins serve as scaffolds for assembling the SCF complex. CrCUL1A was significantly accumulated in ‘Wuzishatangju’ pollen and strongly interacted with CrSKP1-e. These results suggest that CrCUL1A potentially functions as a scaffold for the SCF complex in pollen. However, the *CrCUL1B* gene was less expressed in ‘Wuzishatangju’ pollen. Further studies are necessary to confirm whether *CrCUL1B* is a redundant or an alternative protein for assembling the SCF complex involved in the SI system.

### Identification of putative SCF-type E3 complex in ‘Wuzishatangju’ pollen

The interactions between F-box and SKP1 proteins have been extensively explored. In *Arabidopsis*, 92 AtSLF-S_2_-related proteins were identified; most of them interacted with one or more SKP1 proteins in the yeast system (Wang et al., 2004). To investigate the SCF complex involved in the SI reaction in *P. inflata*, 17 SLF proteins and an SLF-like protein were co-immunoprecipitated with PiSSK1:FLAG:GFP (Li et al., 2016). In apple, MdSSK1 interacted with 4 MdSFBB and 6 MdSFBBL proteins in the yeast system (Yuan et al., 2014). In the present study, yeast system interaction assays revealed that CrSKP1-e protein interacted with 4 CrFBX proteins (CrFBX2, CrFBX9, CrFBX13 and CrFBX15) in ‘Wuzishatangju’ and at least one CrFBX protein (CrFBX7) in ‘Chuntianju’. The LCI and in vitro assays indicated that CrSKP1-e binds to the N-terminal region of both the CrCUL1A and CrCUL1B proteins. The CrSKP1-e protein could act as an adaptor that links CrFBX and CrCUL1 for assembling of the SCF complex in ‘Wuzishatangju’. Interestingly, CrSKP1-e not only interacted with the S-locus CrFBX proteins (CrFBX2, CrFBX7 and CrFBX9) in mandarin but also bound to the non-S-locus CrFBX proteins (CrFBX13, CrFBX15). These results suggest that CrSKP1-e potentially functions as an adaptor in the SI reaction. However, further studies are needed to elucidate the function of *CrSKP1-e* genes in the SI reaction of ‘Wuzishatangju’.

## Conclusions

In summary, the genome-wide characterization of the 298 F-box family proteins was performed using the in silico method based on the *C. clementina* reference genome, which supported in-depth identification of the S-locus *F-box* genes. Ten pollen-specific *CrFBX* genes homologous to SFBB/SLFL are mapped into the S-locus. CrSKP1-e connects the S-locus and non-S-locus CrFBX proteins to the two CrCUL1 proteins (CrCUL1A and CrCUL1B) for SCF complex assembling in ‘Wuzishatangju’ pollen.

##  Supplemental Information

10.7717/peerj.10578/supp-1 Supplemental Information 1 Identification of F-box family proteins in *C. clementina* (A) A total of 12 additional C-terminal domains were obtained by searching Pfam/SMART website and NCBI conserved domain database; (B) 12 groups (group A-group L) were tentatively classified according to the common node. A total of 46 non-redundant FBA subfamily proteins are identified (45 proteins are cluster into group B and one cluster into group E); (C) The majority of FBA subfamily (30 genes) are lack of intron and 14 genes have one intron, only two predicted genes (Ciclev10011950m.g, Ciclev10013681m.g) have two introns.

10.7717/peerj.10578/supp-2 Supplemental Information 2 Expression analyses of FBA subfamily genes. Expression patterns of FBA subfamily genes were performed in different tissues (leaf, petal, filament, pollen, stigma, style and ovary) of ‘Wuzishatangju’ by RT-PCR analyses.

10.7717/peerj.10578/supp-3 Supplemental Information 3 Expression analyses of FBA subfamily genes. Verification of expression patterns of *CrFBX1- CrFBX 17* in different tissues of ‘Wuzishattangju’ by qRT-PCR analyses.

10.7717/peerj.10578/supp-4 Supplemental Information 4 Sequence alignment of *CrFBX7* gene between ‘Wuzishatangju’ and ‘Chuntianju’ A 1-bp deletion (red star) in 3′-termini of CrFBX7 in ‘Wuzishatangju’ is predicted to cause failure of translation termination. *CrFBX7* derived from ‘Chuntianju’ encodes normal F-box protein.

10.7717/peerj.10578/supp-5 Supplemental Information 5 The schematic of S-locus in *Citrus* (A) The schematic was described according to the published genome (https://www.citrusgenomedb.org/) and the S-RNase was marked red, *S*_*m*_ *-RNase* (red words) is identified in *C. clementina* and *C. sinensis*; (B) Cglg001950 is identified as *S*_6_ *-RNase* and co-separated with the *S*_6_ genotype of F1 hybrids (Liang et al., 2020).

10.7717/peerj.10578/supp-6 Supplemental Information 6 Characterization of *SKP1* family genes in *C. clementina* (A) Phylogenomic analyses and structure of *SKP1* family genes in the reference genome of *C. clementina*, the *SKP1* family genes were clustered into group I-group Ⅲ; (B) Orthologs of *CrSKP1* in *C. clementina*. *CcSKP1-1* to *CcSKP1-5* were not identified in the transcriptome dataset of *C. reticulata*. *CcSKP1-4* was just annotated as a fragmentary coding sequence without the AUG in *C. clementina* (presented by asterisk); (C) The expression profile of *CrSKP1-e* and *CcSKP1-3* in different tissues of *C. reticulata*. But the *CcSKP1-3* was not detected; (D) The expression profile of *CcSKP1-5* in *C. reticulata*; (E) The expression profile of *CcSKP1-2* in *C. reticulata*. (F) The expression profile of *CcSKP1-1* in *C. reticulata*.

10.7717/peerj.10578/supp-7 Supplemental Information 7 Identification of Cullin (CUL) family proteins in *C. clementina* The orthologs of AtCUL1 were bold. The UniProt accessions followed with corresponding proteins.

10.7717/peerj.10578/supp-8 Supplemental Information 8 Sequence alignment of *CUL1* genes Identity of *CrCUL1A* between *C. reticulata* and *C. clementina* Ciclev10019010m and Ciclev10004406m are peptide accession numbers of *C. clementina* which is downloaded from JGI database (https://phytozome.jgi.doe.gov). The sequence alignment was conducted by DNAMAN software.

10.7717/peerj.10578/supp-9 Supplemental Information 9 Sequence alignment of *CUL1* genes Identity of *CrCUL1B* between *C. reticulata* and *C. clementina*. Ciclev10019010m and Ciclev10004406m are peptide accession numbers of *C. clementina* which is downloaded from JGI database (https://phytozome.jgi.doe.gov). The sequence alignment was conducted by DNAMAN software.

10.7717/peerj.10578/supp-10 Supplemental Information 10 Primer pairs used for expression analyses and cloning of Cullin1 family genes in ’Wuzishatangju’ (*C. reticulata*)

10.7717/peerj.10578/supp-11 Supplemental Information 11 Pollen-specific F-box genes between *C. reticulata* and *C. clementina*

10.7717/peerj.10578/supp-12 Supplemental Information 12 Accession number of Cullin and SKP1 family proteins in NCBI GenBank

10.7717/peerj.10578/supp-13 Supplemental Information 13 Characterization of Cullin1 family genes in *C. clementina*

10.7717/peerj.10578/supp-14 Supplemental Information 14 Raw data from expression analyses of CrFBX1-16, CrSKP1, and CrCUL1 and LUC assays

10.7717/peerj.10578/supp-15 Supplemental Information 15 AGel electrophoresis images for pollen-specific F-box gene identification; gel electrophoresis images for semi-quantitative PCR analyses of CrCULA and CrCULB gene; raw WB figures for Pull-down assays; and raw gel electrophoresis images for CrSKP1-e and Cc

## Additional Information and Declarations

### Data Availability

The following information was supplied regarding data availability: The raw measurements are available in the Supplemental Files. Additional data is available at the following Figshare repositories: - Ren, Yi (2020): Raw data_dataset1.rar. figshare. Dataset. https://doi.org/10.6084/m9.figshare.11786199.v1. - Ren, Yi (2020): Raw data_Figure.rar. figshare. Figure. https://doi.org/10.6084/m9.figshare.11783730.v1. The resequencing data of ’Wuzishatangju’ is available at Figshare: Ren, Yi (2020): wuzi.rmdup.bam. figshare. Dataset. https://doi.org/10.6084/m9.figshare.11880303.v1
Pollen and pistil transcriptome datasets of ‘Wuzishatangju’ are available at Figshare: Ren, Yi (2020): Citrus_reticulata_Blanco-Unigene.5-3.fa. figshare. Dataset. https://doi.org/10.6084/m9.figshare.12198627.v1.
