# Supplementary figures and images for: SKP1-like protein, CrSKP1-e, interacts with pollen-specific F-box proteins and assembles into SCF-type E3 complex in ‘Wuzishatangju’ (Citrus reticulata Blanco) pollen
Source: PeerJ. 2020 Dec 22;8:e10578. doi: 10.7717/peerj.10578 (PMC7761267; doi:10.7717/peerj.10578)

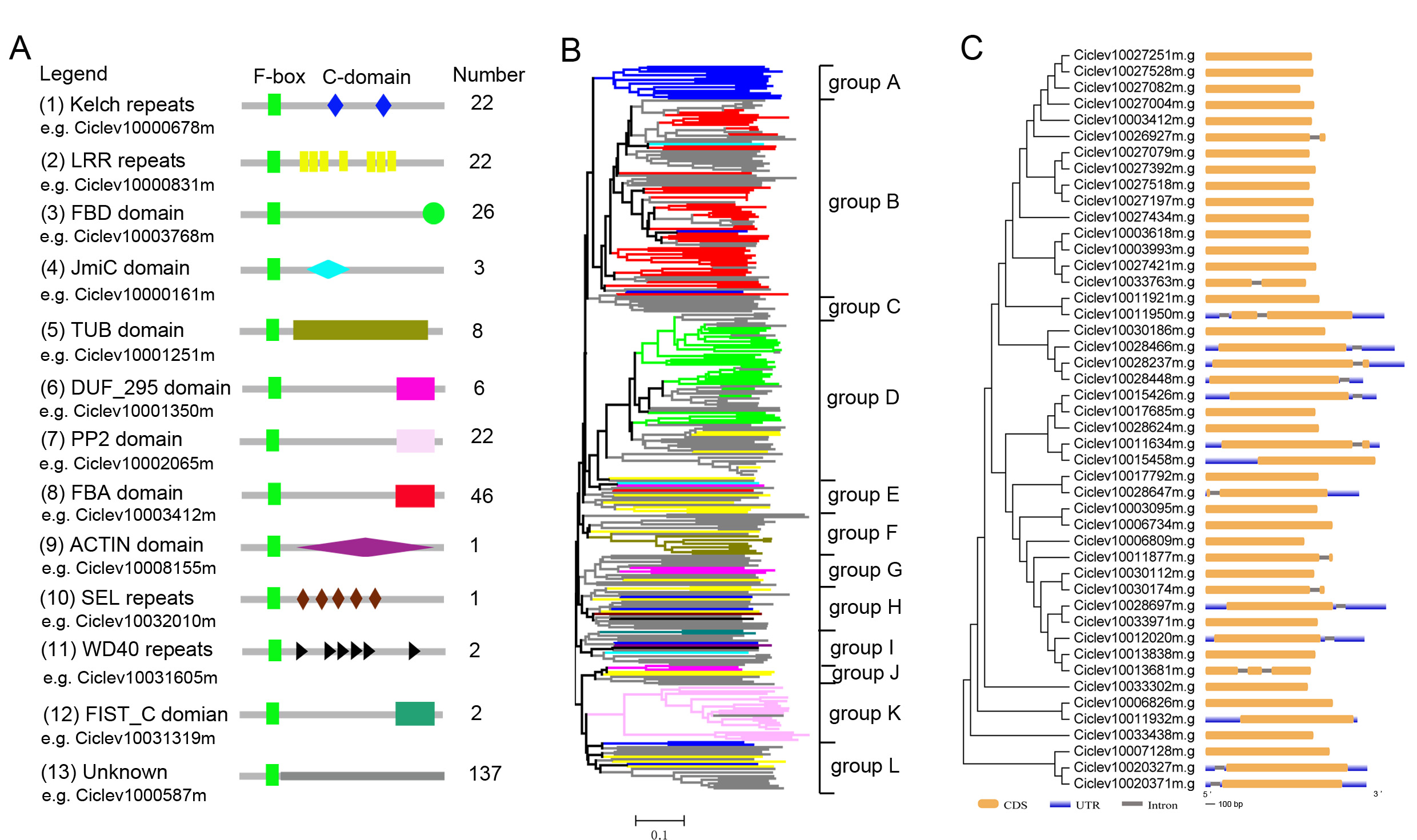

Supplement: Supplemental Information 1 — (A) A total of 12 additional C-terminal domains were obtained by searching Pfam/SMART website and NCBI conserved domain database; (B) 12 groups (group A-group L) were tentatively classified according to the common node. A total of 46 non-redundant FBA subfamily proteins are identified (45 proteins are cluster into group B and one cluster into group E); (C) The majority of FBA subfamily (30 genes) are lack of intron and 14 genes have one intron, only two predicted genes (Ciclev10011950m.g, Ciclev10013681m.g) have two introns. [file peerj-08-10578-s001.png]

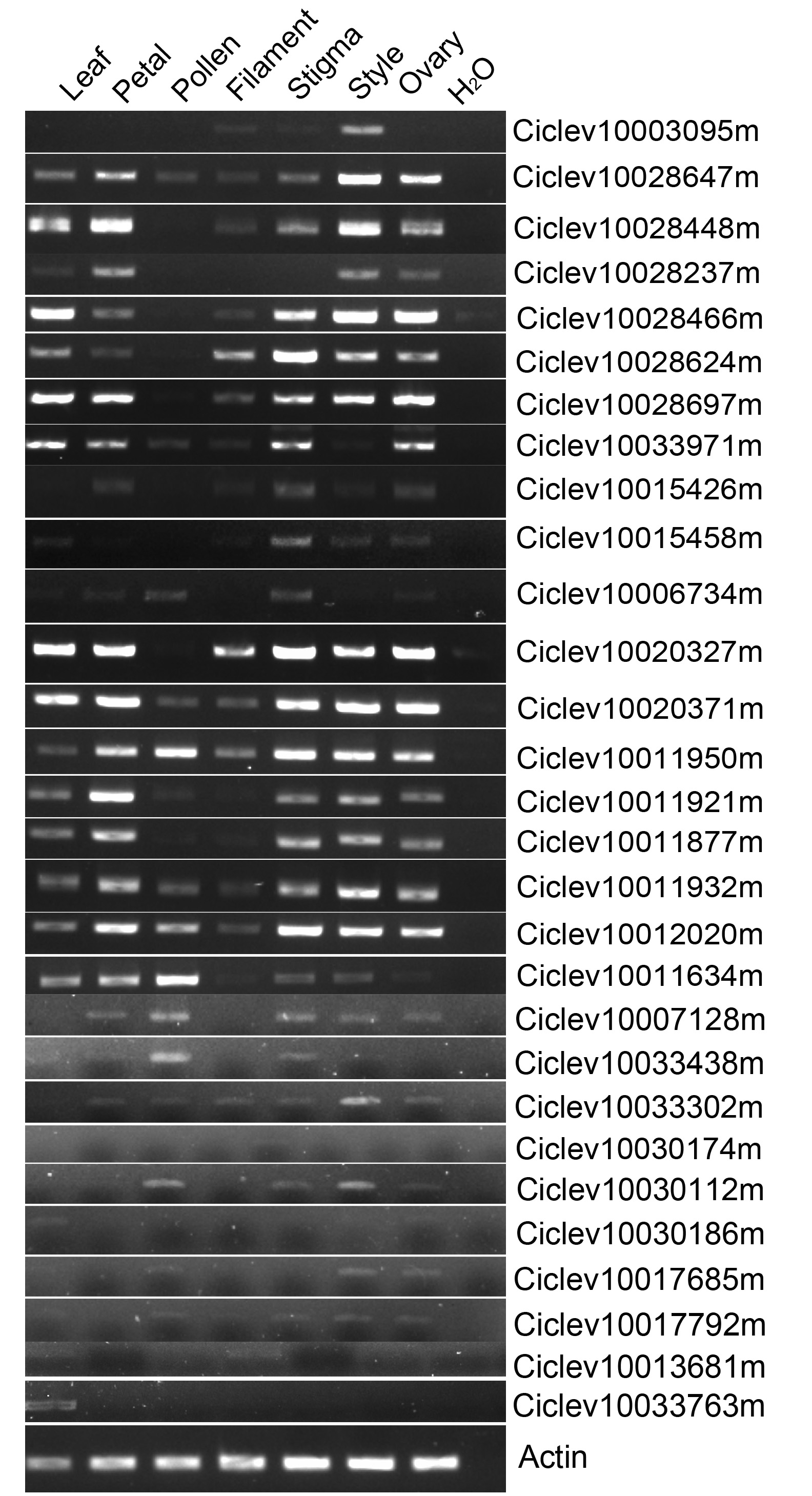

Supplement: Supplemental Information 2 — Expression patterns of FBA subfamily genes were performed in different tissues (leaf, petal, filament, pollen, stigma, style and ovary) of ‘Wuzishatangju’ by RT-PCR analyses. [file peerj-08-10578-s002.png]

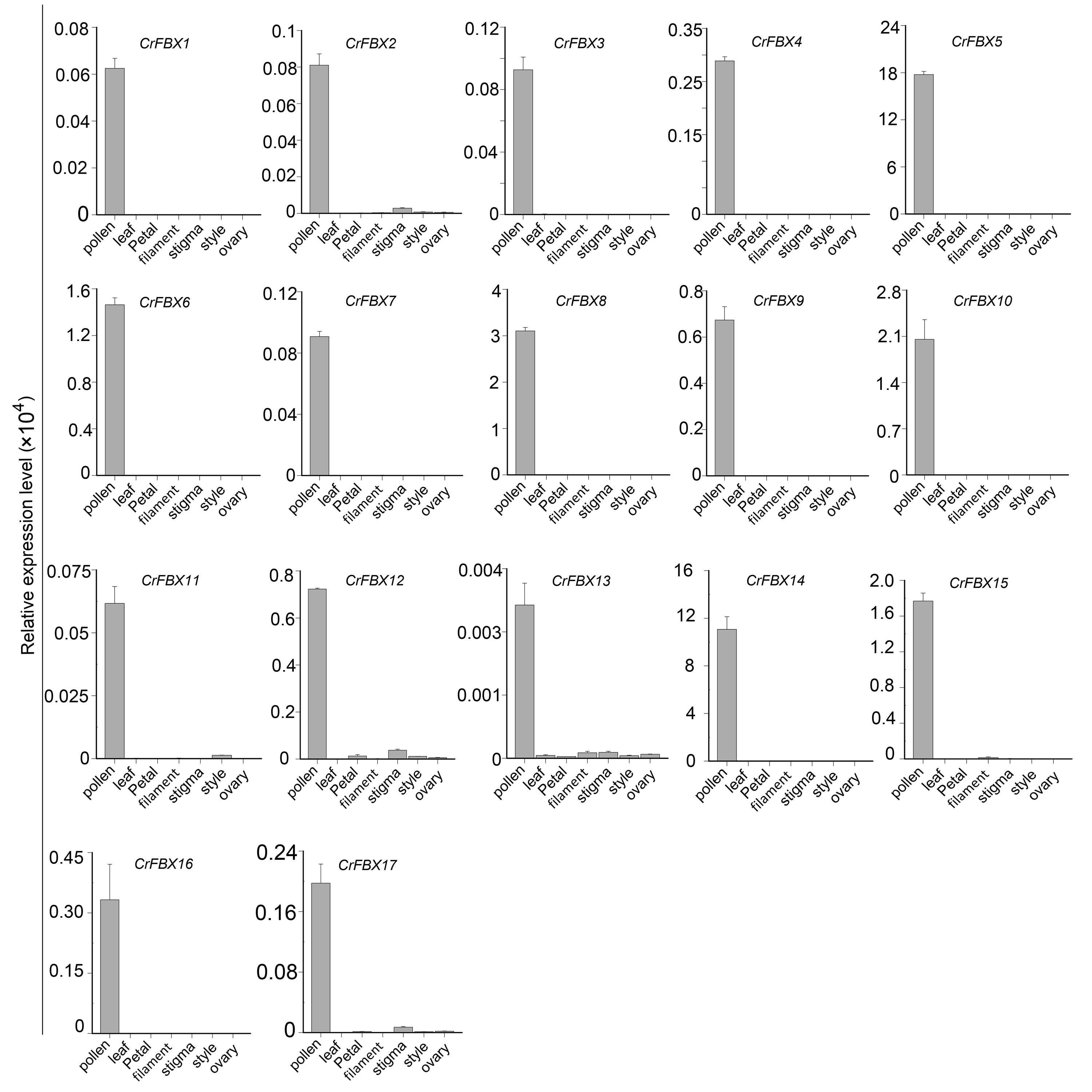

Supplement: Supplemental Information 3 — Verification of expression patterns of CrFBX1- CrFBX 17 in different tissues of ‘Wuzishattangju’ by qRT-PCR analyses. [file peerj-08-10578-s003.png]

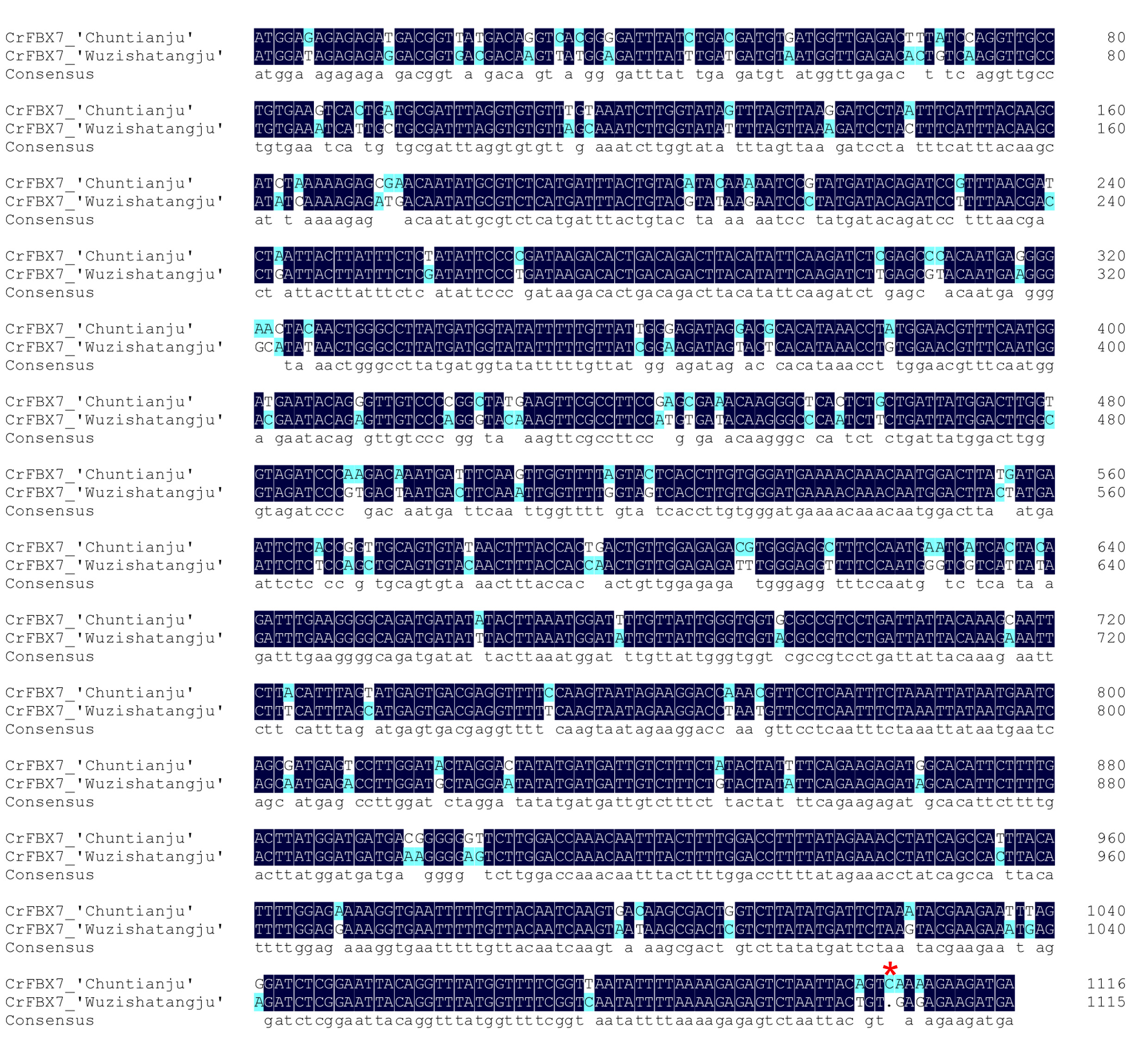

Supplement: Supplemental Information 4 — A 1-bp deletion (red star) in 3′-termini of CrFBX7 in ‘Wuzishatangju’ is predicted to cause failure of translation termination. CrFBX7 derived from ‘Chuntianju’ encodes normal F-box protein. [file peerj-08-10578-s004.png]

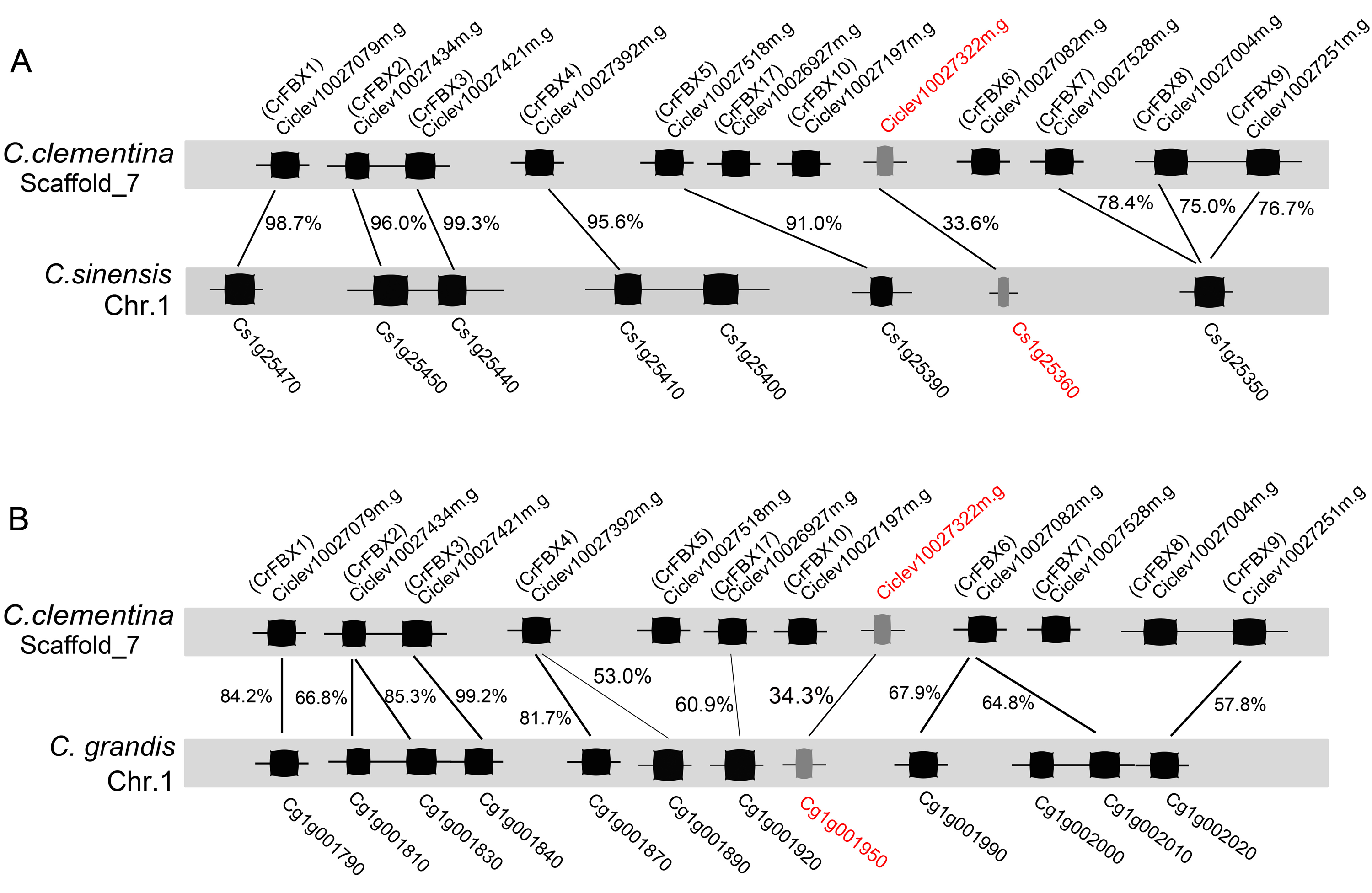

Supplement: Supplemental Information 5 — (A) The schematic was described according to the published genome (https://www.citrusgenomedb.org/) and the S-RNase was marked red, Sm -RNase (red words) is identified in C. clementina and C. sinensis; (B) Cglg001950 is identified as S6 -RNase and co-separated with the S6 genotype of F1 hybrids (Liang et al., 2020). [file peerj-08-10578-s005.png]

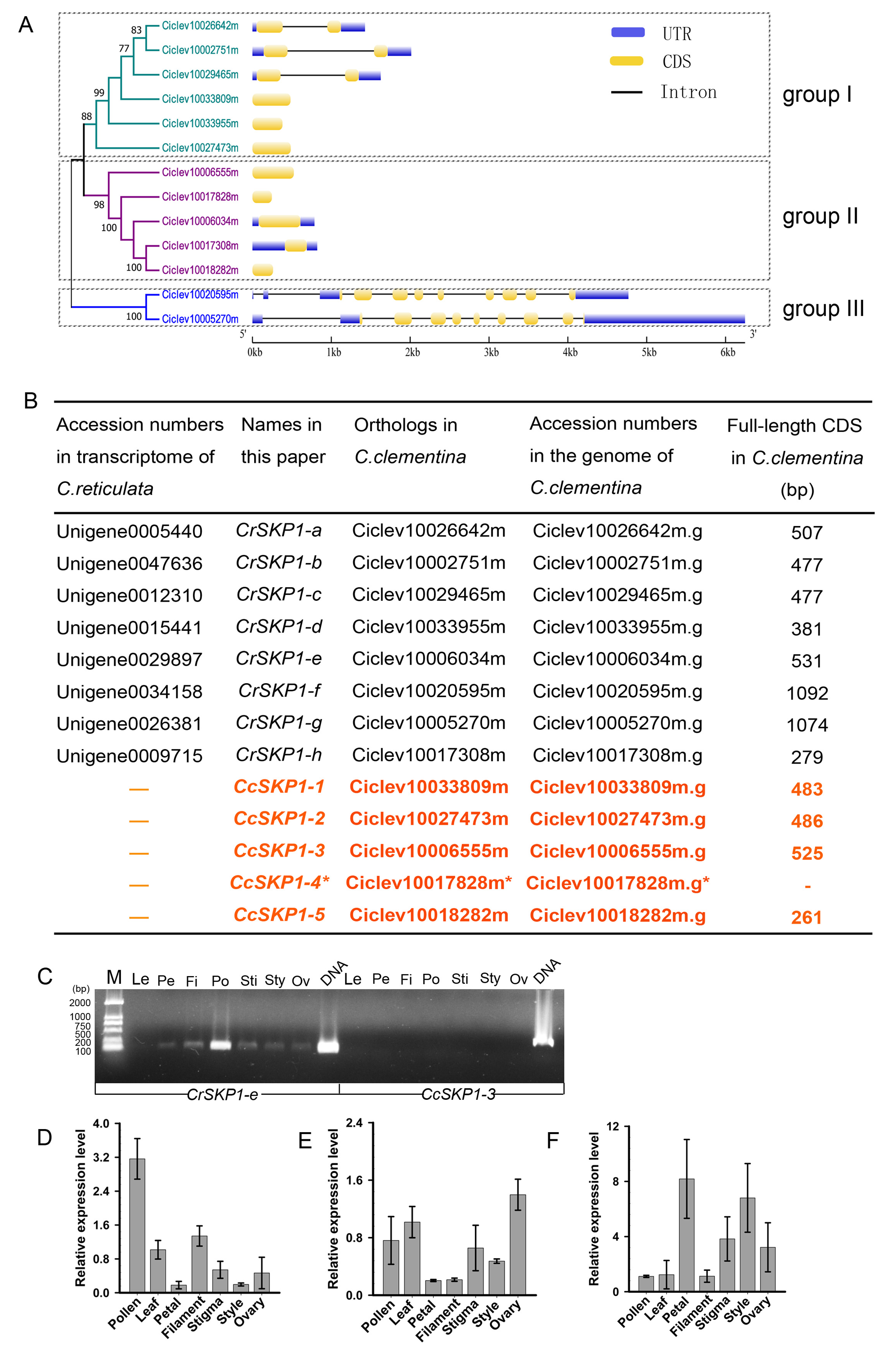

Supplement: Supplemental Information 6 — (A) Phylogenomic analyses and structure of SKP1 family genes in the reference genome of C. clementina, the SKP1 family genes were clustered into group I-group Ⅲ; (B) Orthologs of CrSKP1 in C. clementina. CcSKP1-1 to CcSKP1-5 were not identified in the transcriptome dataset of C. reticulata. CcSKP1-4 was just annotated as a fragmentary coding sequence without the AUG in C. clementina (presented by asterisk); (C) The expression profile of CrSKP1-e and CcSKP1-3 in different tissues of C. reticulata. But the CcSKP1-3 was not detected; (D) The expression profile of CcSKP1-5 in C. reticulata; (E) The expression profile of CcSKP1-2 in C. reticulata. (F) The expression profile of CcSKP1-1 in C. reticulata. [file peerj-08-10578-s006.png]

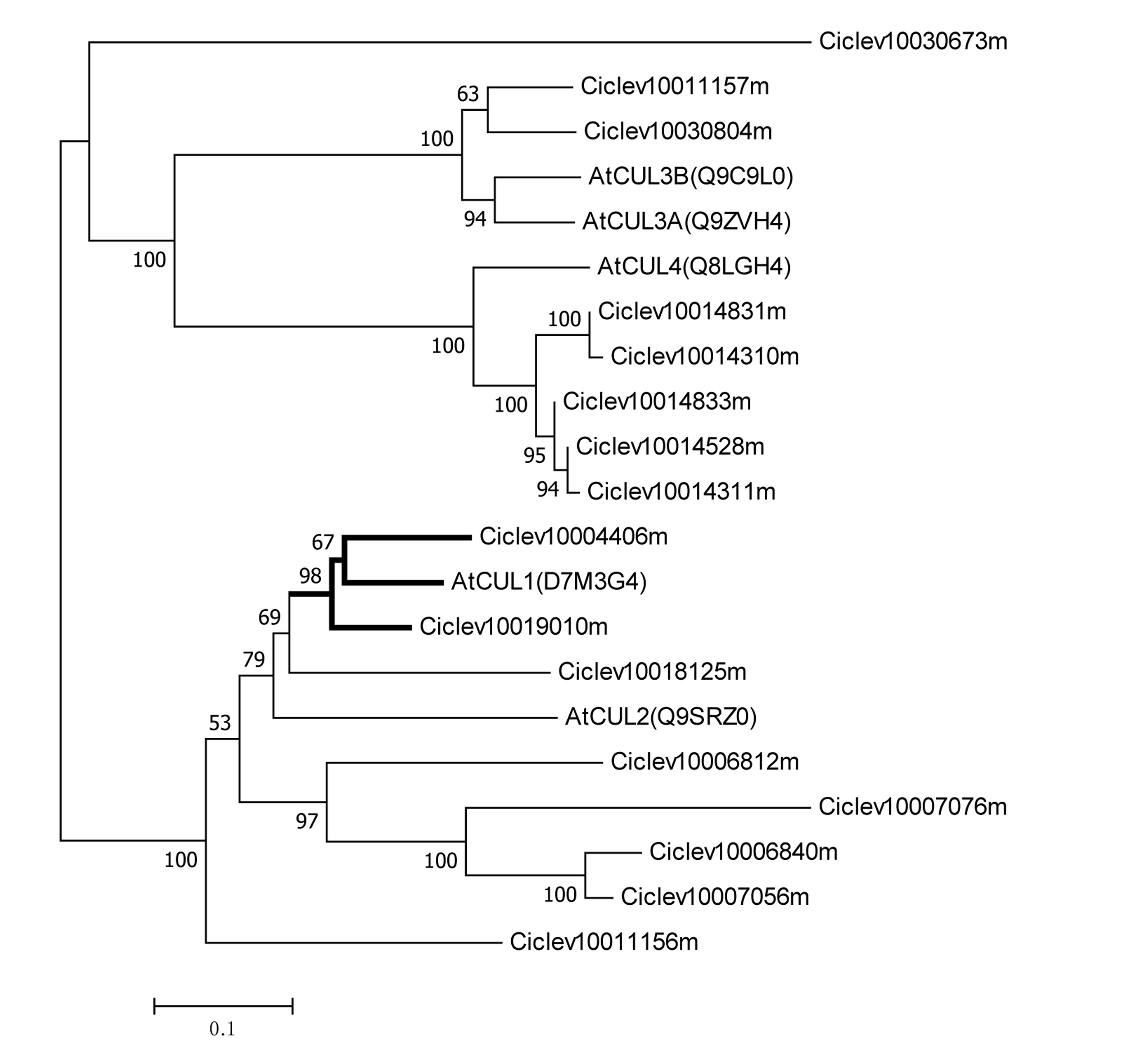

Supplement: Supplemental Information 7 — The orthologs of AtCUL1 were bold. The UniProt accessions followed with corresponding proteins. [file peerj-08-10578-s007.jpg]

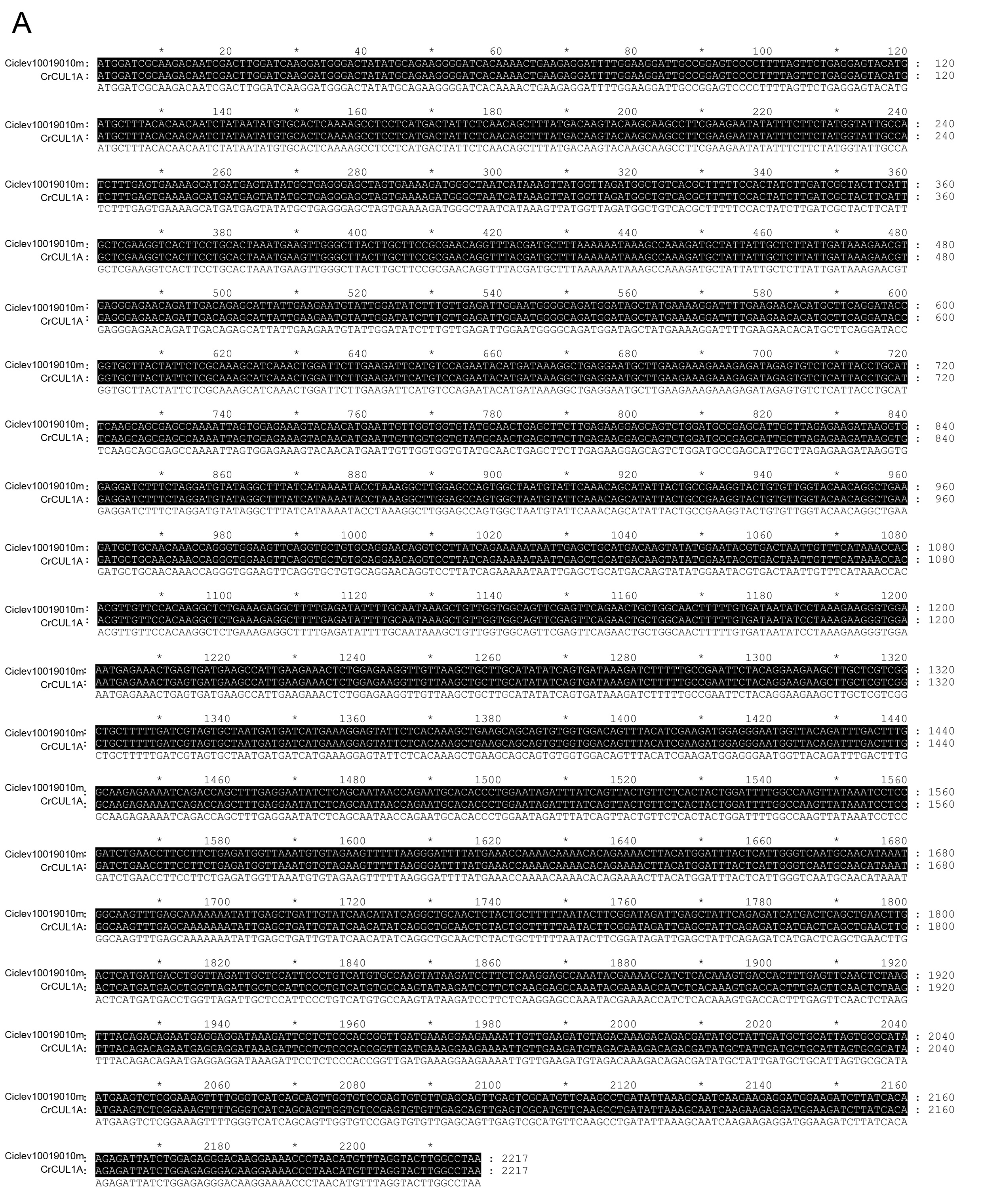

Supplement: Supplemental Information 8 — Identity of CrCUL1A between C. reticulata and C. clementina Ciclev10019010m and Ciclev10004406m are peptide accession numbers of C. clementina which is downloaded from JGI database (https://phytozome.jgi.doe.gov). The sequence alignment was conducted by DNAMAN software. [file peerj-08-10578-s008.png]

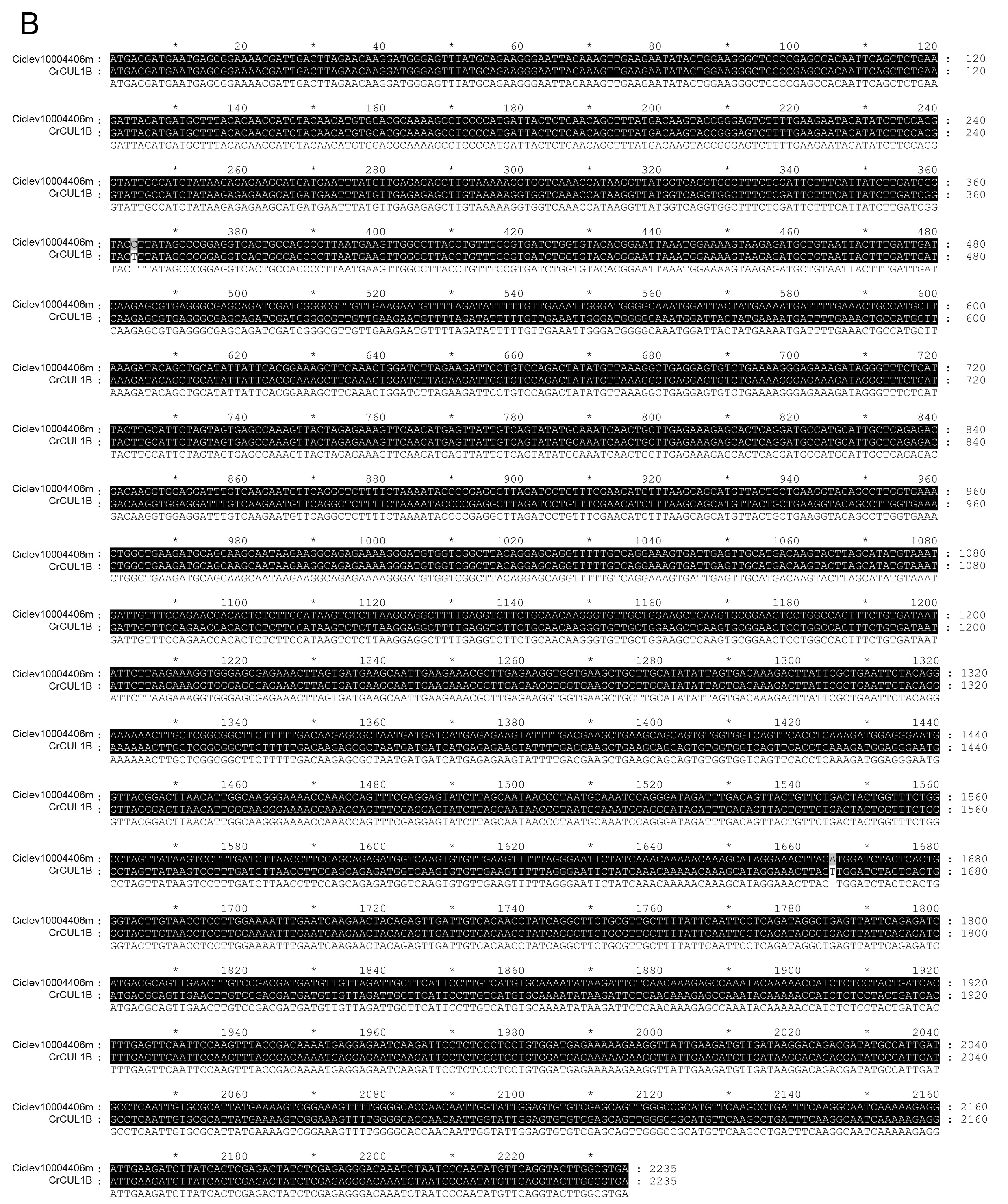

Supplement: Supplemental Information 9 — Identity of CrCUL1B between C. reticulata and C. clementina. Ciclev10019010m and Ciclev10004406m are peptide accession numbers of C. clementina which is downloaded from JGI database (https://phytozome.jgi.doe.gov). The sequence alignment was conducted by DNAMAN software. [file peerj-08-10578-s009.png]

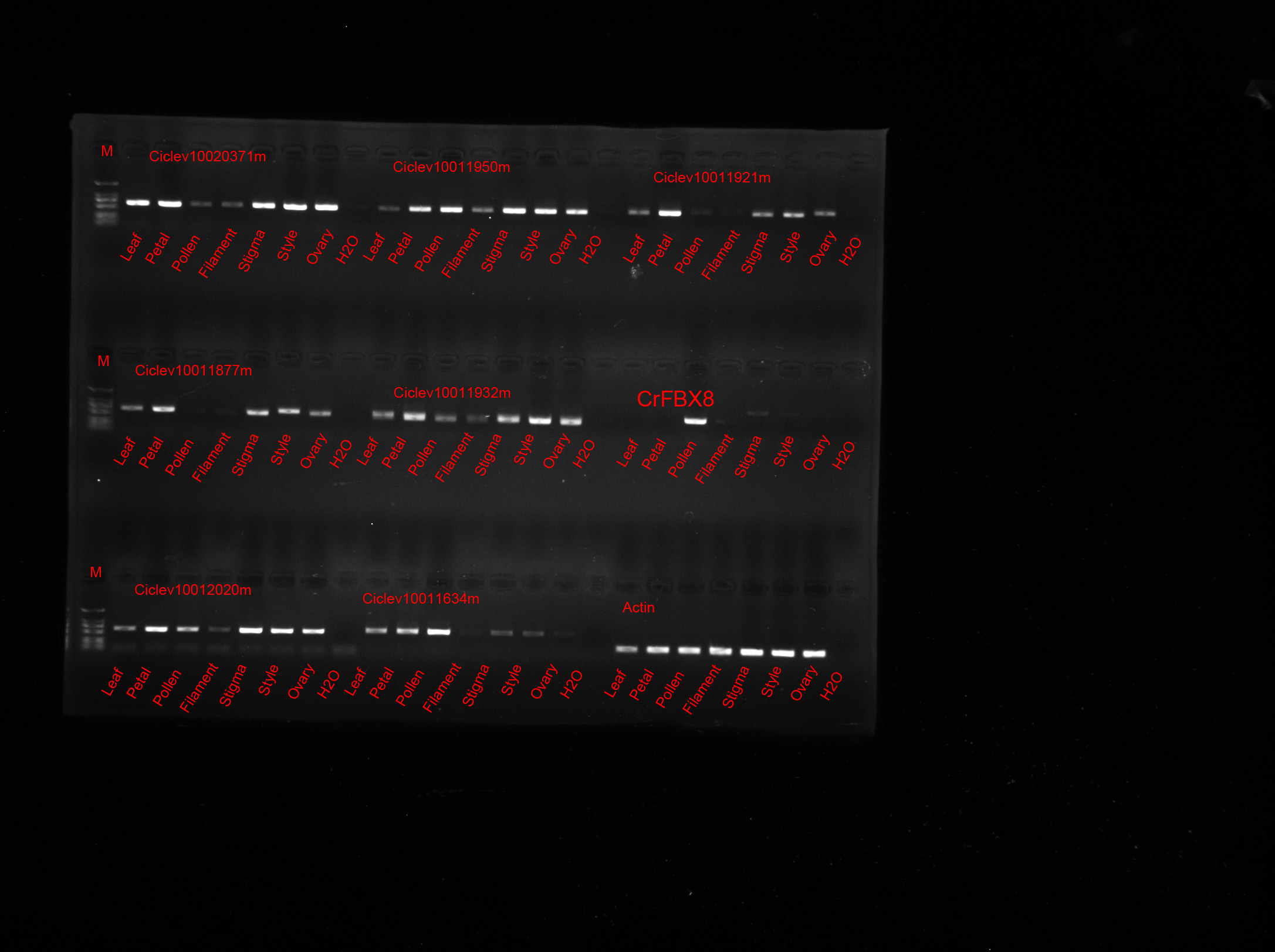

Supplement: Supplemental Information 15 [file peerj-08-10578-s015.zip › Raw data_Figure/Raw data_F-box _PCR results/F-box identification_Uncorpped gels_1.jpg]

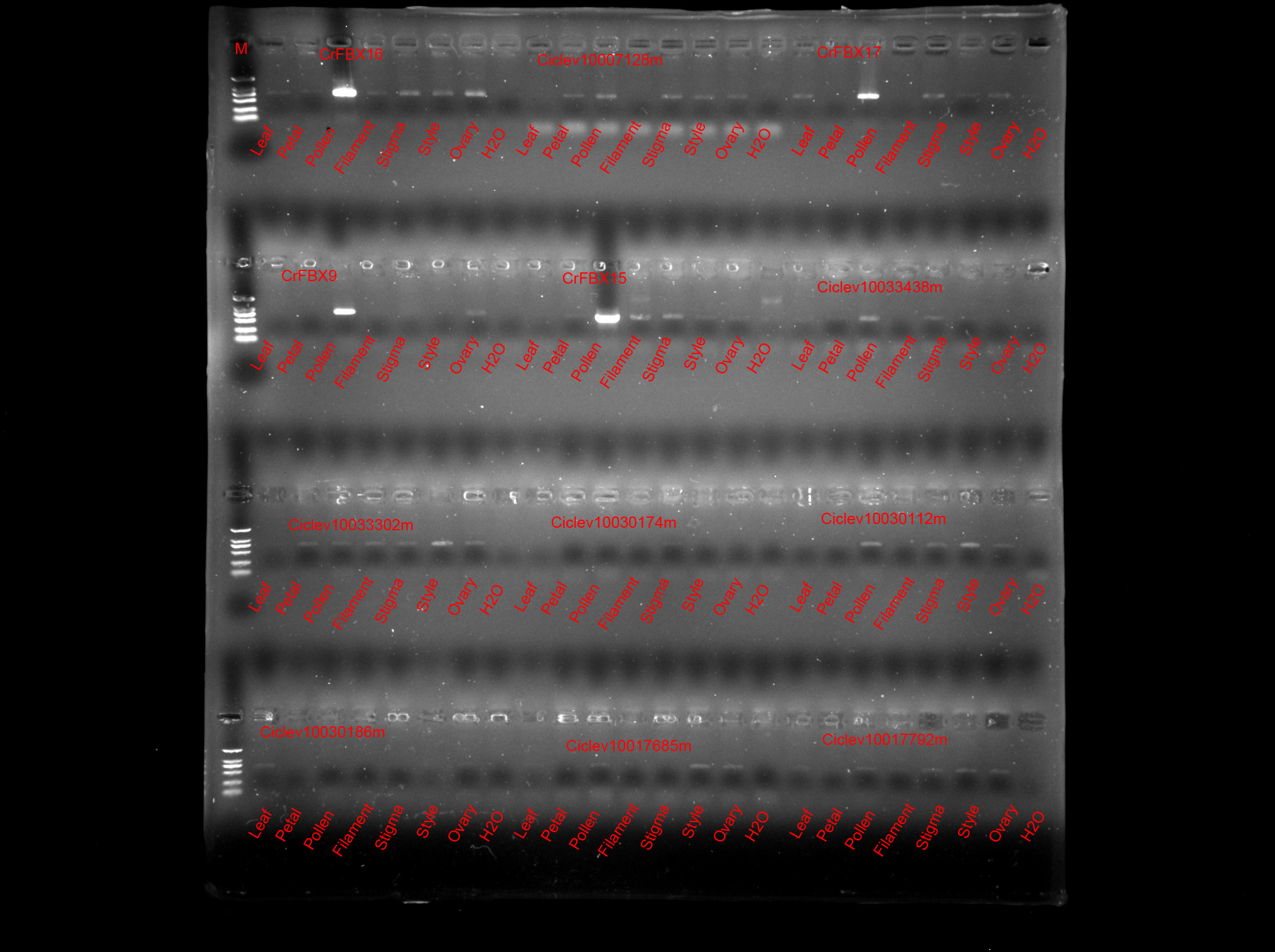

Supplement: Supplemental Information 15 [file peerj-08-10578-s015.zip › Raw data_Figure/Raw data_F-box _PCR results/F-box identification_Uncorpped gels_2.jpg]

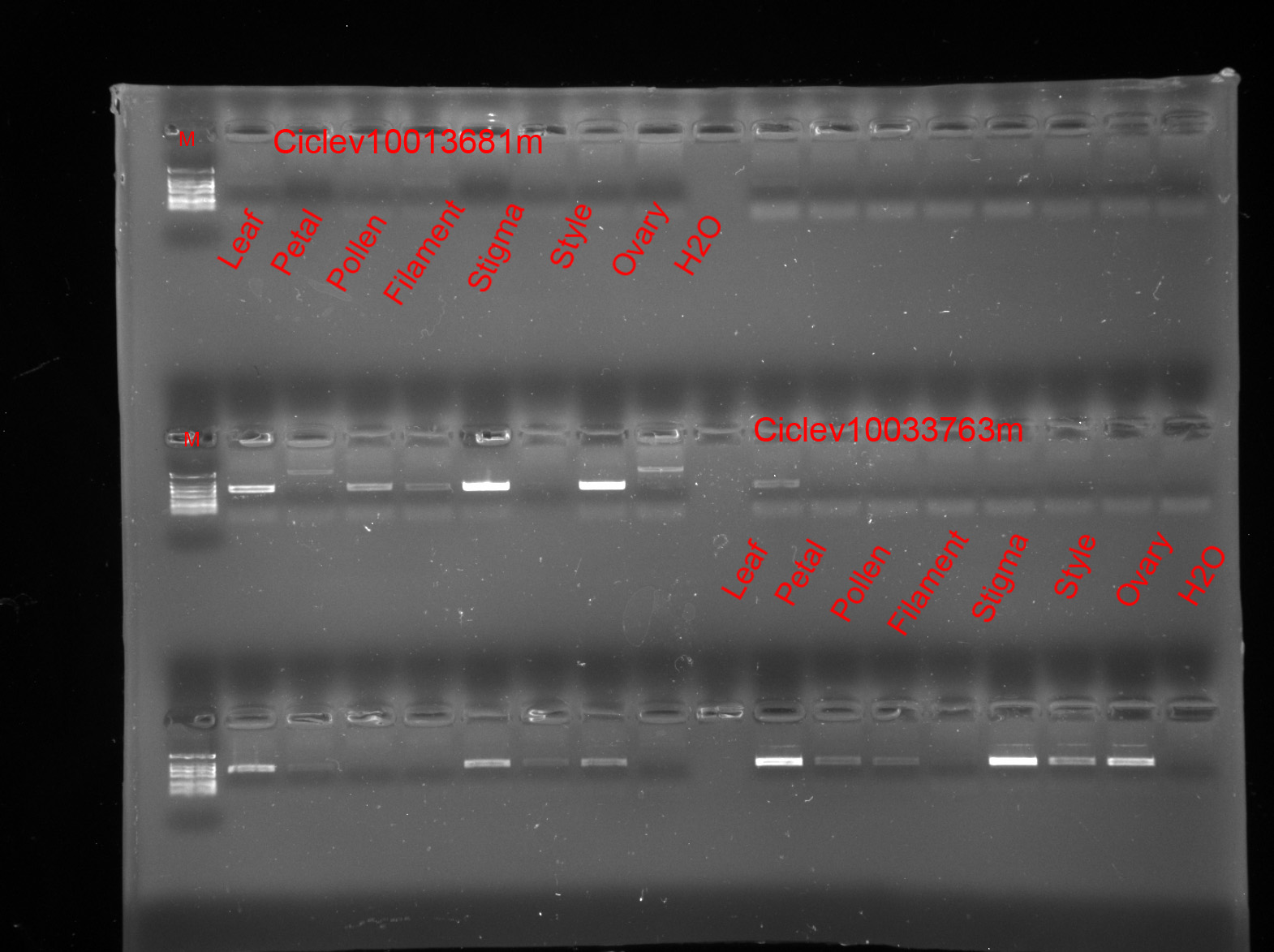

Supplement: Supplemental Information 15 [file peerj-08-10578-s015.zip › Raw data_Figure/Raw data_F-box _PCR results/F-box identification_Uncorpped gels_3.jpg]

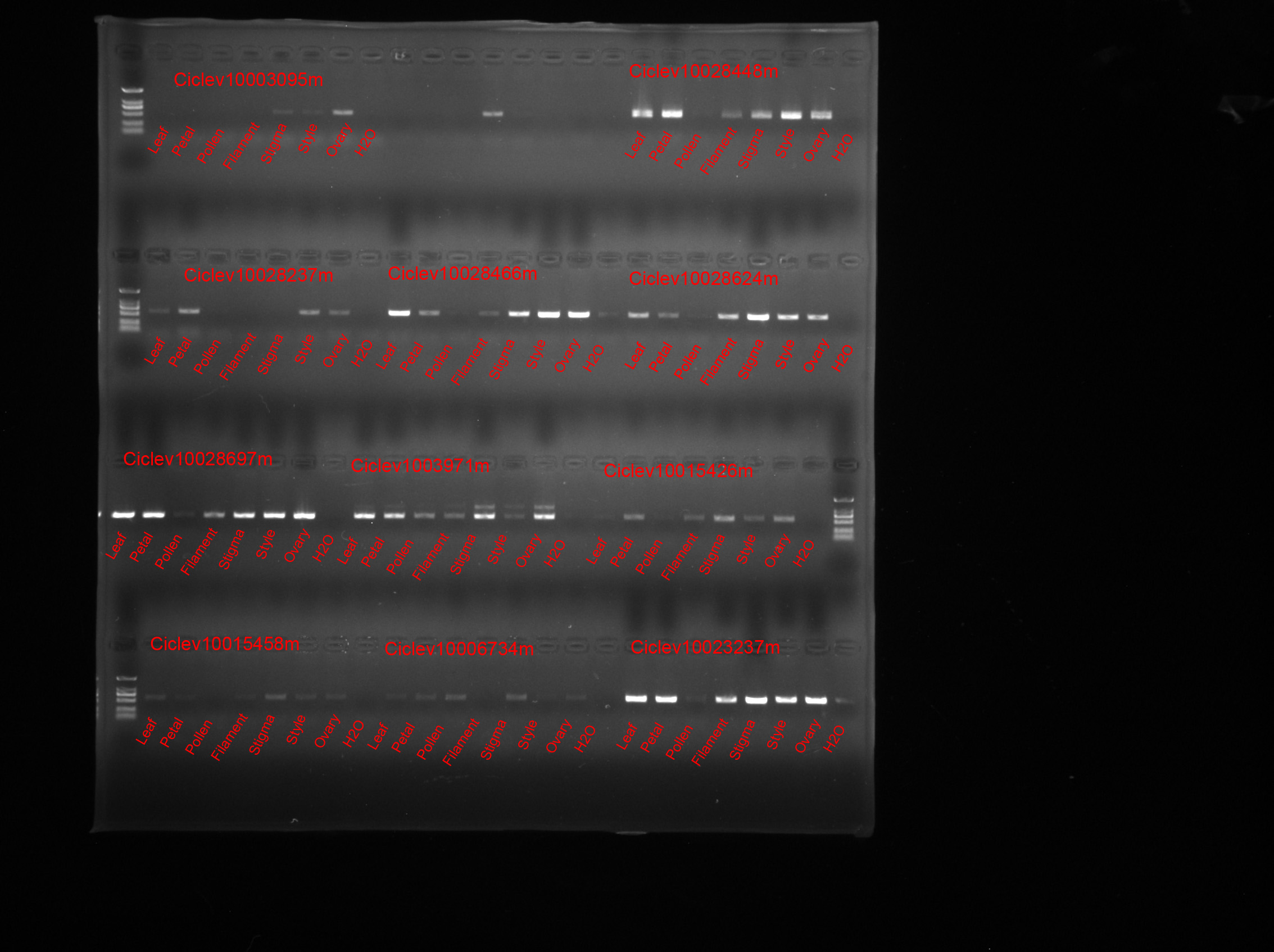

Supplement: Supplemental Information 15 [file peerj-08-10578-s015.zip › Raw data_Figure/Raw data_F-box _PCR results/F-box identification_Uncorpped gels_4.jpg]

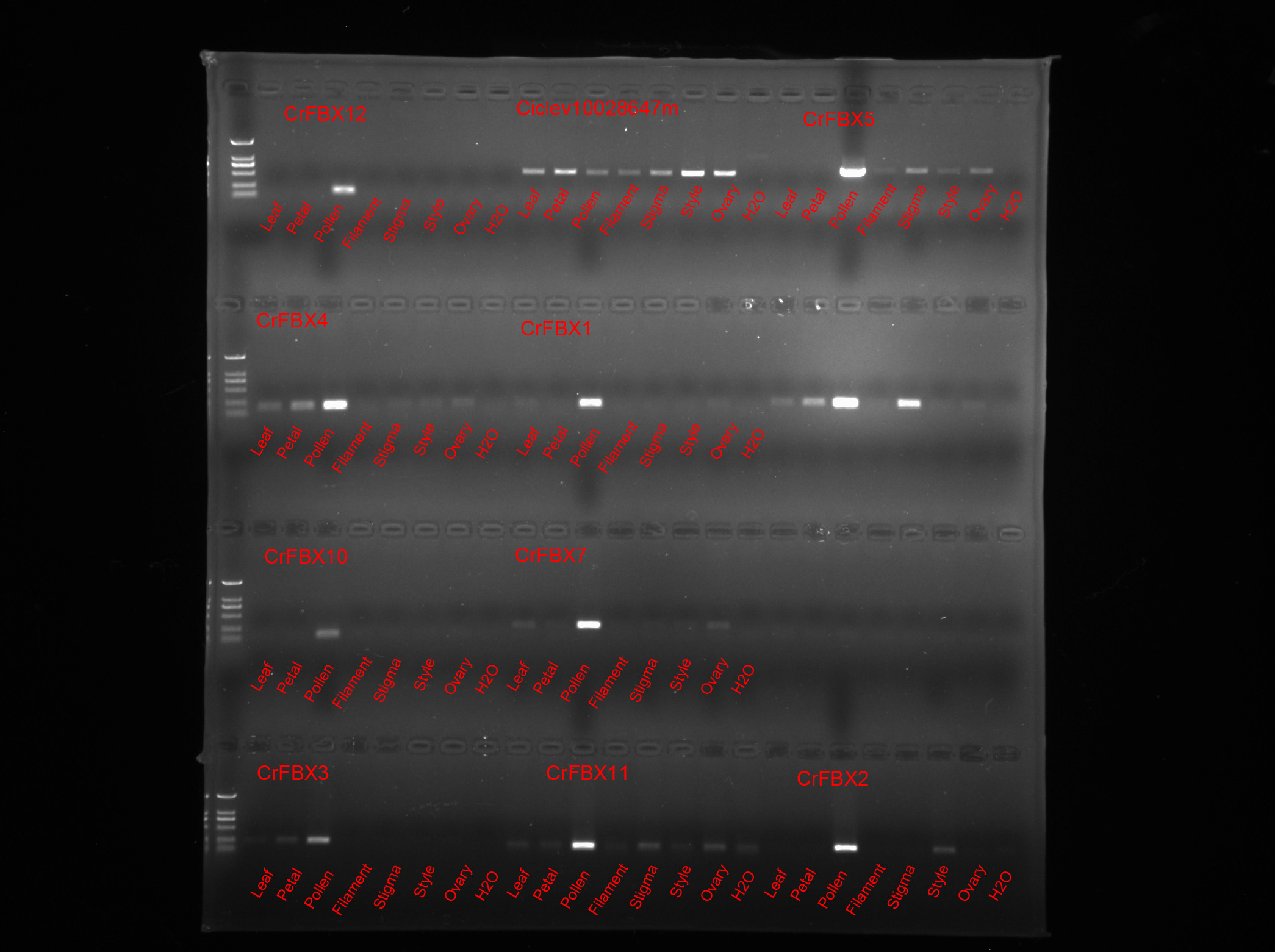

Supplement: Supplemental Information 15 [file peerj-08-10578-s015.zip › Raw data_Figure/Raw data_F-box _PCR results/F-box identification_Uncorpped gels_5.jpg]

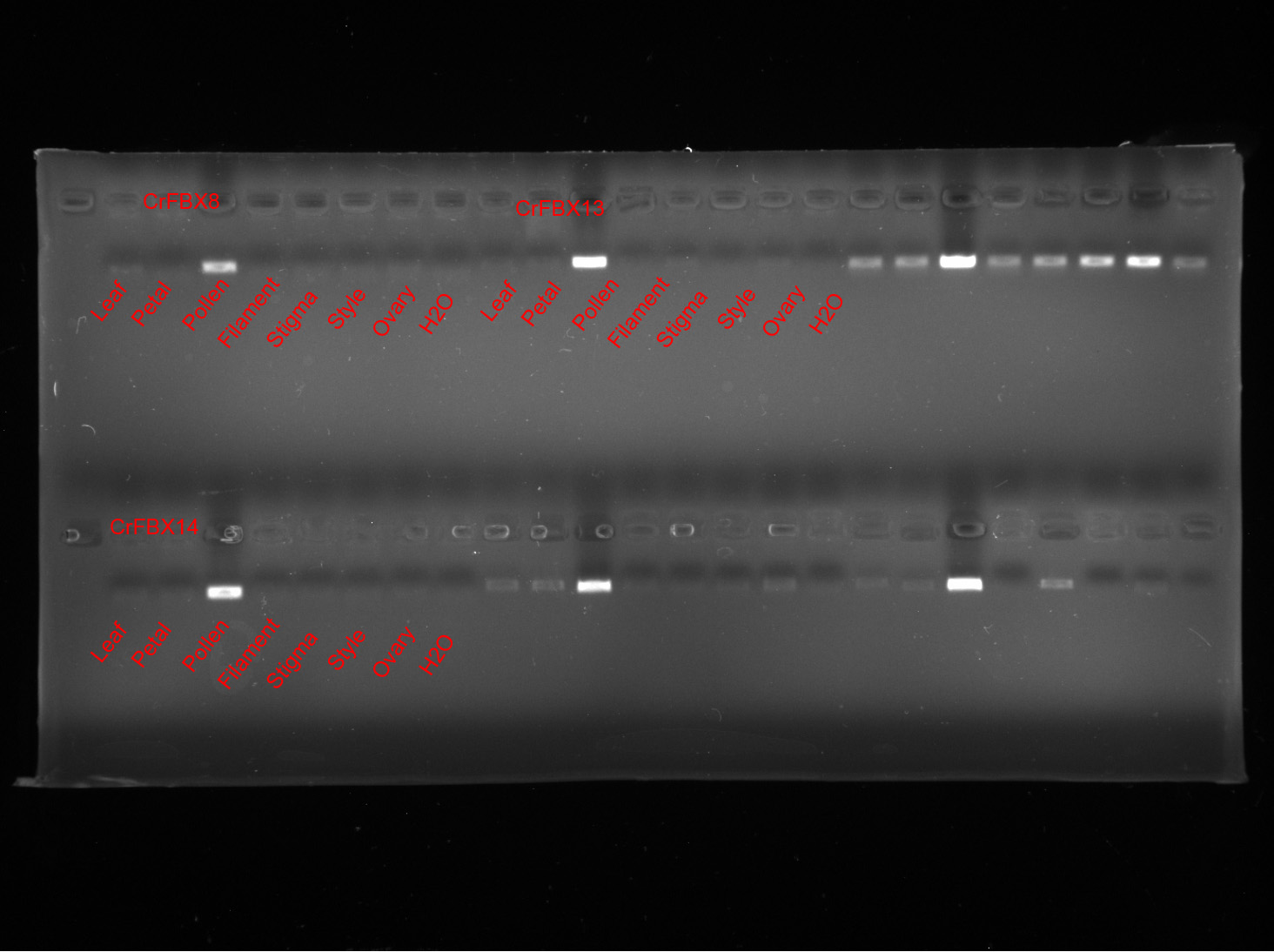

Supplement: Supplemental Information 15 [file peerj-08-10578-s015.zip › Raw data_Figure/Raw data_F-box _PCR results/F-box identification_Uncorpped gels_6.jpg]

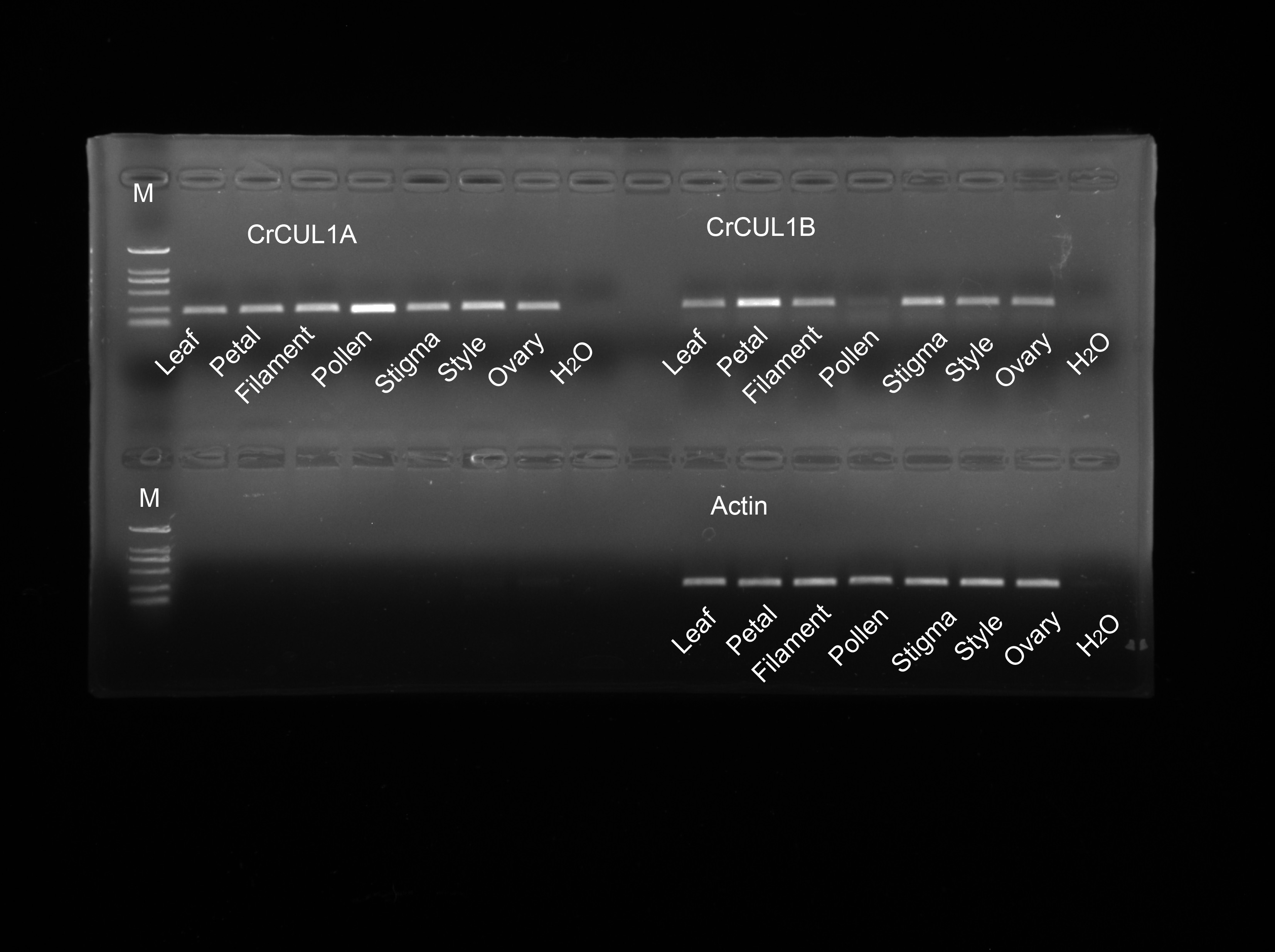

Supplement: Supplemental Information 15 [file peerj-08-10578-s015.zip › Raw data_Figure/Raw data_Figure 4/Figure 4_uncropped gel.jpg]

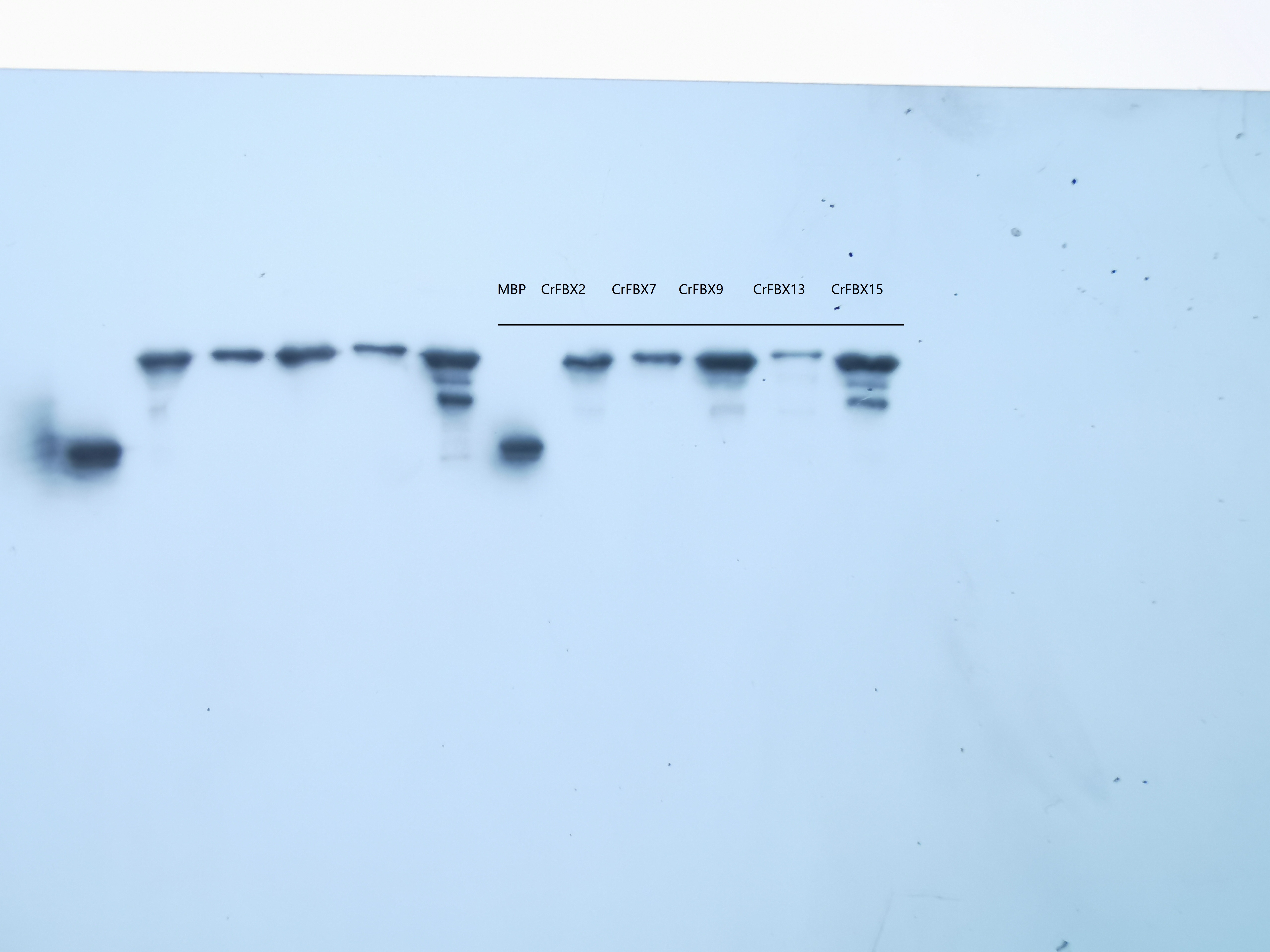

Supplement: Supplemental Information 15 [file peerj-08-10578-s015.zip › Raw data_Figure/Raw data_pull down test/Fig. 5B_input.jpg]

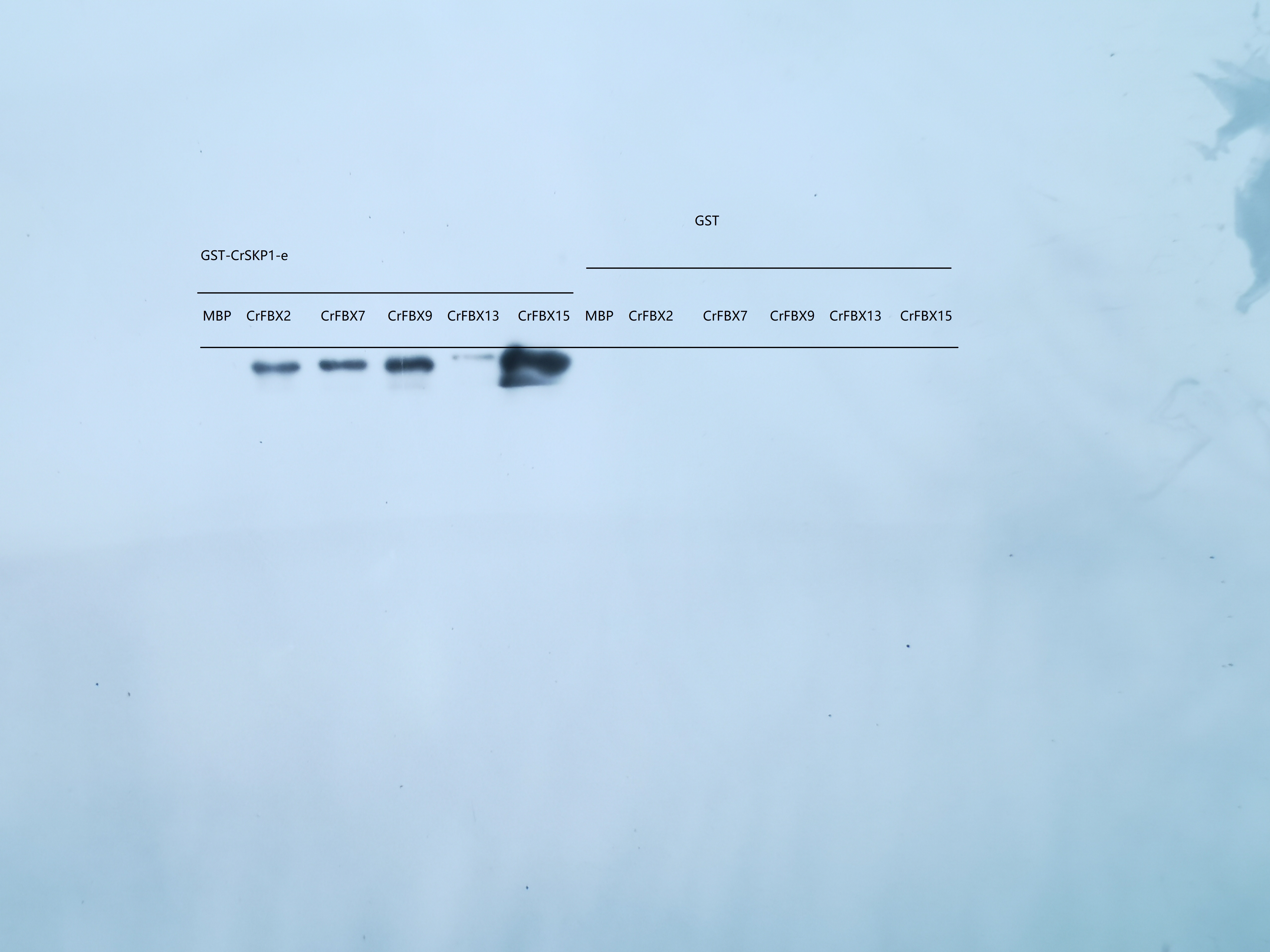

Supplement: Supplemental Information 15 [file peerj-08-10578-s015.zip › Raw data_Figure/Raw data_pull down test/Fig. 5B_pull down.jpg]

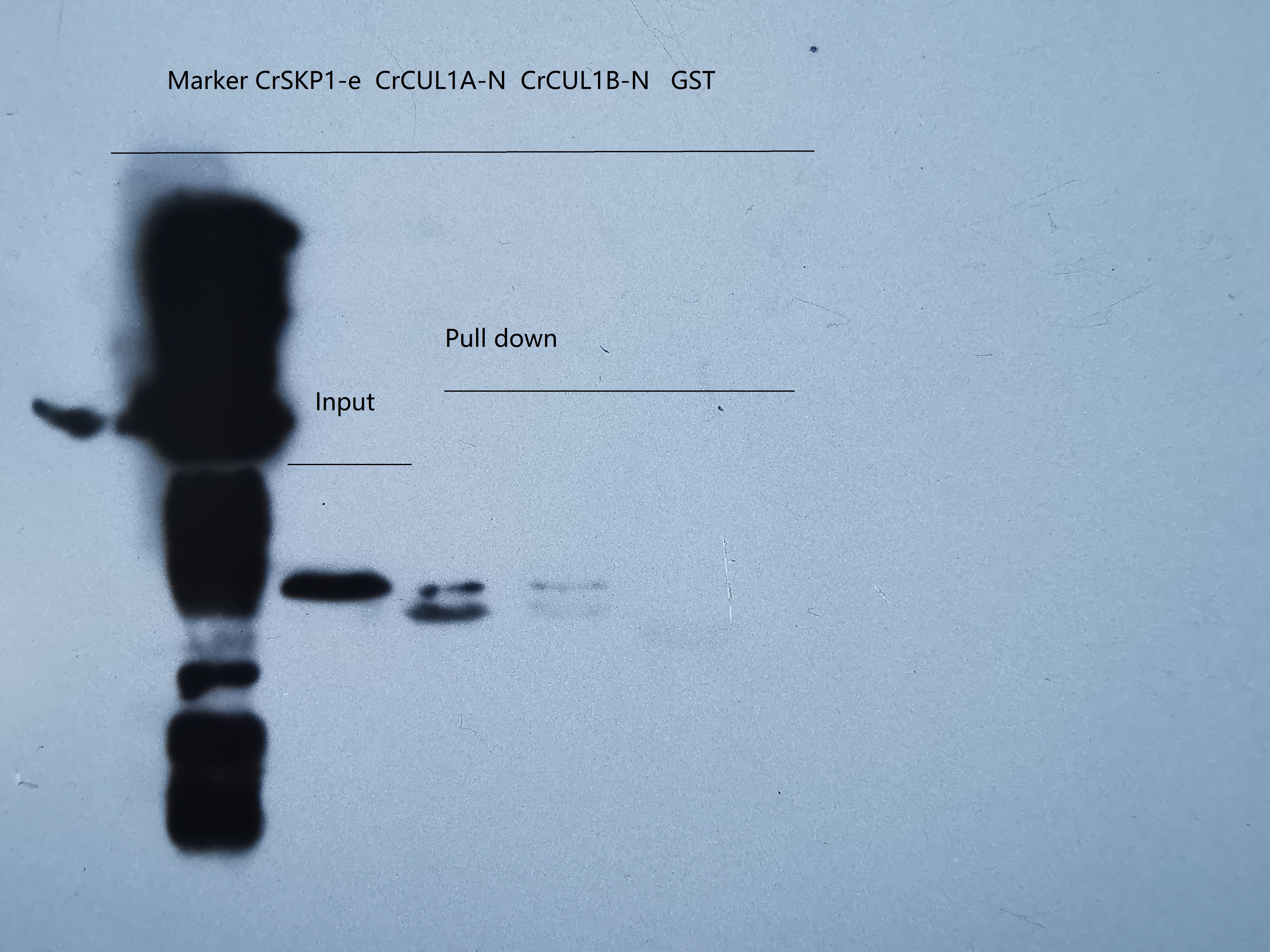

Supplement: Supplemental Information 15 [file peerj-08-10578-s015.zip › Raw data_Figure/Raw data_pull down test/Fig. 6E.jpg]

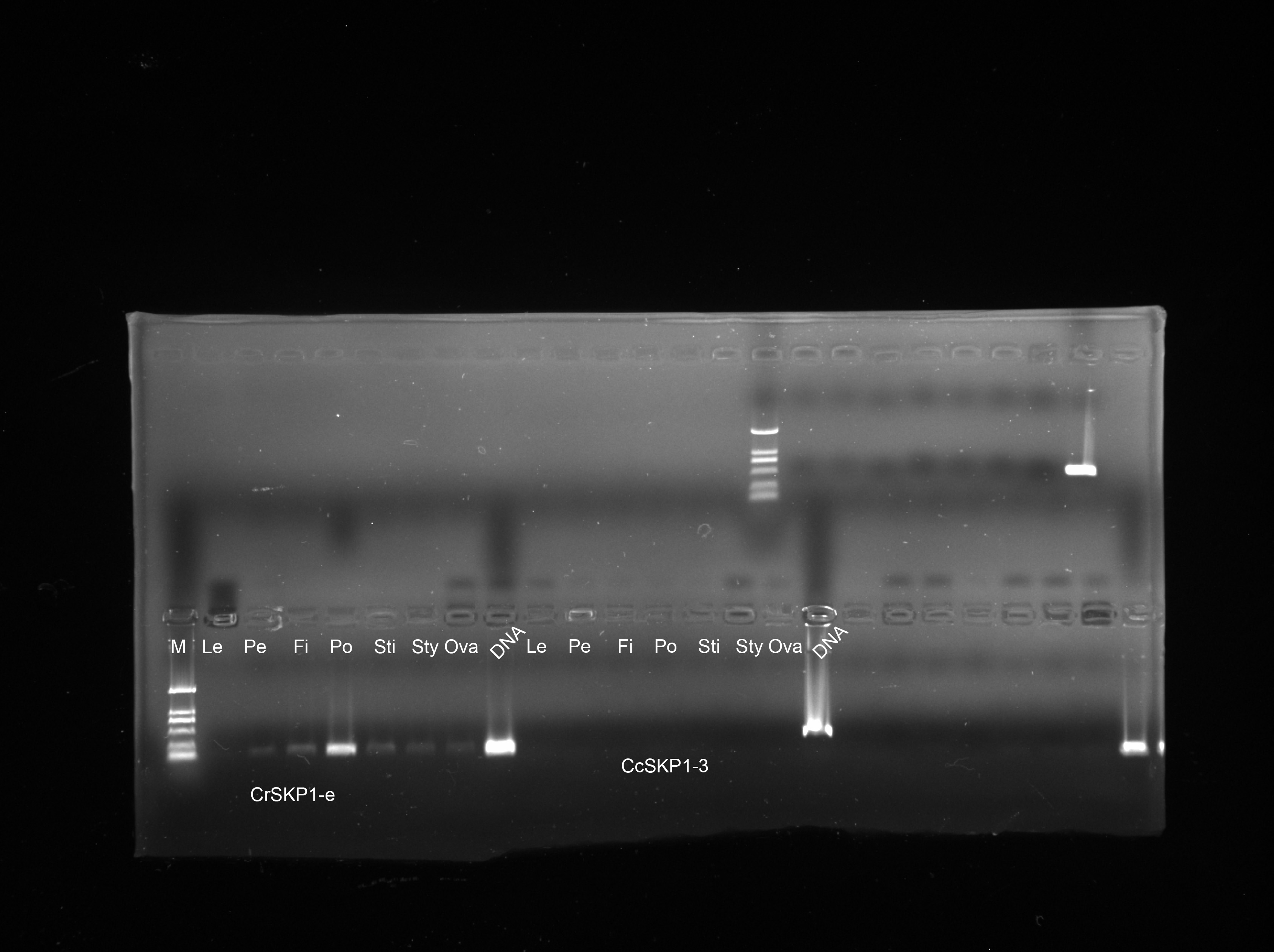

Supplement: Supplemental Information 15 [file peerj-08-10578-s015.zip › Raw data_Figure/Raw data_Supplemental Figure 3/Supplemental Figure 3_uncropped gel.jpg]
